# Transcription factor NTL9 negatively regulates Arabidopsis vascular cambium development during stem secondary growth

**DOI:** 10.1093/plphys/kiac368

**Published:** 2022-08-11

**Authors:** Hiroki Sugimoto, Tomoko Tanaka, Nobuhiko Muramoto, Ritsuko Kitagawa-Yogo, Norihiro Mitsukawa

**Affiliations:** Toyota Central R&D Laboratories, Inc., Nagakute, Aichi 480-1192, Japan; Toyota Central R&D Laboratories, Inc., Nagakute, Aichi 480-1192, Japan; Toyota Central R&D Laboratories, Inc., Nagakute, Aichi 480-1192, Japan; Toyota Central R&D Laboratories, Inc., Nagakute, Aichi 480-1192, Japan; Toyota Central R&D Laboratories, Inc., Nagakute, Aichi 480-1192, Japan

## Abstract

In plant stems, secondary vascular development is established through the differentiation of cylindrical vascular cambium, producing secondary xylem (wood) and phloem (bast), which have economic importance. However, there is a dearth of knowledge on the genetic mechanism underlying this process. NAC with Transmembrane Motif 1-like transcription factor 9 (NTL9) plays a central role in abiotic and immune signaling responses. Here, we investigated the role of NTL9 in vascular cambium development in Arabidopsis (*Arabidopsis thaliana*) inflorescence stems by identifying and characterizing an Arabidopsis *phloem circular-timing* (*pct*) mutant. The *pct* mutant exhibited enhanced vascular cambium formation following secondary phloem production. In the *pct* mutant, although normal organization in vascular bundles was maintained, vascular cambium differentiation occurred at an early stage of stem development, which was associated with increased expression of cambium-/phloem-related genes and enhanced cambium activity. The *pct* mutant stem phenotype was caused by a recessive frameshift mutation that disrupts the transmembrane (TM) domain of NTL9. Our results indicate that NTL9 functions as a negative regulator of cambial activity and has a suppressive role in developmental transition to the secondary growth phase in stem vasculature, which is necessary for its precise TM domain-mediated regulation.

## Teaser

NAC with Transmembrane Motif 1-like transcription factor 9 (NTL9) is a negative regulator of cambial activity and suppresses developmental transition to the secondary growth phase in stem vasculature.

## Introduction

Stem secondary vascular growth in seed plants, especially woody species, is fundamental for structural integrity, along with long-distance transport of water, nutrients, and signaling molecules essential for their growth and fitness (Spicer and Groover, 2010; Miyashima et al., 2013; Agustí and Blázquez, 2020). This developmental process is also indispensable for increasing terrestrial biomass production and carbon sink function (carbon sequestration), thereby allowing the utilization of renewable and sustainable resources, such as wood, bast, latex, and fuels (Spicer and Groover, 2010; Nieminen et al., 2012; Tan et al., 2017). The industrial demand for such materials calls for cellular engineering to enhance secondary vascular growth (i.e. proliferation, differentiation, and patterning).

Arabidopsis (*Arabidopsis thaliana*) is an annual herbaceous plant that exhibits extensive secondary vascular growth in roots, hypocotyl, and inflorescence stems (Altamura et al., 2001; Chaffey et al., 2002; Sehr et al., 2010; Mazur et al., 2014; Nieminen et al., 2015). An apical-basal developmental series is observed in inflorescence stems with the tip regions exhibiting only primary growth and the basal regions near the rosette base showing extensive secondary growth (Altamura et al., 2001; Sehr et al., 2010). Furthermore, phloem fibers, secondary rays, and storied cambial cells—typical features of woody plant stems—are found in inflorescence stems (Mazur and Kurczynska, 2012; MacMillan et al., 2013). Thus, Arabidopsis is an appropriate model for investigating the molecular frameworks of stem secondary vascular growth, which facilitates the identification of key regulators conserved in trees (Strabala and Macmillan, 2013; Barra-Jiménez and Ragni, 2017).

Stem vascular growth is a highly ordered developmental process that can be divided into two phases: primary and secondary development (Spicer and Groover, 2010; Miyashima et al., 2013; Agustí and Blázquez, 2020). Primary stems have multiple disconnected vascular bundles arranged collaterally and circumferentially, and in each bundle, the procambium derived from the apical meristem is located between the xylem and phloem. At an early stage of stem secondary growth, while the procambium develops into the fascicular cambium, the interfascicular parenchyma cells or starch sheath cells (the innermost cortical cell layer) between vascular bundles proliferate and transdifferentiate into interfascicular cambium. Interfascicular cambium formation progresses laterally and interconnects the fascicular cambia in two adjacent vascular bundles, leading to the closing of the ring of meristematic activity, and in turn, the formation of a vascular cambium in a concentric cylinder form. Finally, the vascular cambium provides cells for secondary xylem toward the center of the stem and secondary phloem toward the periphery, enabling an increase in plant girth (Sehr et al., 2010; Miyashima et al., 2013; Mazur et al., 2014; Fischer et al., 2019). Thus, the establishment of vascular cambium through de novo formation of interfascicular cambium is the primary process driving secondary growth.

Despite its importance, studies on the temporal and spatial regulation of vascular cambium establishment are lacking. Vascular cambium is formed within “troublesome” thick stem organs that limit direct observational and anatomical approaches. Further, forward genetic screening for mutants with defects in timing of initiation of meristematic activity is especially challenging and requires laborious procedures (Wunderling et al., 2017). Few genes participating in the initiation of secondary growth, such as *continuous vascular ring* (*cov1*; Parker et al., 2003), *high cambial activity* (*hca*; Pineau et al., 2005), and *high cambial activity2* (*hca2*; Guo et al., 2009), as well as *more lateral growth1* and *reduced in lateral growth1* (*mol1* and *rul1*; Agusti et al., 2011; Gursanscky et al., 2016), have been identified in mutants with enhanced interfascicular cambia formation.

The NAC family (NAM, ATAF1/2, and CUC2) is one of the largest plant-based transcription factor families (Kim et al., 2007; Mohanta et al., 2020). Among the NAC proteins, 13 members (NTL1–4 and NTL6–14), termed as NAC with Transmembrane Motif 1-like (NTL) family of transcription factors, have highly conserved NAC domains and α-helical transmembrane (TM) domains in their N-terminal and distant C-terminal regions, respectively, and are classified as membrane-bound transcription factors (MTFs; Kim et al., 2007; Seo et al., 2008; Liang et al., 2015). Among the 13 NTLs, NTL9 (also known as a calmodulin-binding NAC protein, CBNAC) is likely (yet still controversial) localized in the endoplasmic reticulum (Kim et al., 2007; Yoon et al., 2008; Block et al., 2014; Liang et al., 2015; Zheng et al., 2015; Guo et al., 2017). Osmotic stress has been reported to trigger NTL9 release from the membrane, allowing it to function as a signal transducer for this stress signaling pathway (Yoon et al., 2008). NTL9 is also a key regulator of developmental leaf senescence and modulates the expression of a series of *senescence-associated genes* (*SAGs*) via epigenetic control by the histone H3 lysine 27 tri-methylation demethylase, RELATIVE OF EARLY FLOWERING6 (REF6; Yoon et al., 2008; Wang et al., 2019). Furthermore, NTL9 transcriptionally regulates defense- and salicylic acid (SA) biosynthesis-related genes, including *pathogenesis-related protein 1* (*PR1*) and *isochorismate synthase 1* (*ICS1*), and plays an essential role in plant innate immune responses, such as induction of effector-triggered immunity and stomatal immunity (Mukhtar et al., 2011; Kim et al., 2012; Block et al., 2014; Zheng et al., 2015; Guo et al., 2017). Thus, NTL9 is a key mediator of the crosstalk between intrinsic and extrinsic environmental cues.

Considering the involvement of NTL9 as a key hub of developmental senescence, abiotic stress, and the plant immune system, we investigated the role of NTL9 and its splice variants in vascular cambium development during stem secondary growth. Using a frameshift mutation in *NTL9* in Arabidopsis inflorescence stems, we investigated phenotypic changes in stem secondary growth as well as differentiation of interfascicular cambium and secondary phloem. Therefore, our study illustrates a mechanism of regulation of vascular cambium development at the genetic and structural levels.

## Results

### Isolation of *phloem circular-timing* (*pct*) mutant exhibiting enhanced secondary growth of inflorescence stems

We first identified an original mutant from T-DNA insertion line populations that were designated as *pct.o*, which exhibited increased levels of secondary growth in the primary inflorescence stems, as described in detail in the next section following phenotypic analysis (Supplemental Figure S1). Segregation analysis indicated that the inflorescence stem phenotype of the mutant was recessive and segregated independently of the transgene insertion, indicating that the mutant phenotype was not caused by a T-DNA insertional mutation and/or its transgene expression (Supplemental Table S1). Therefore, we obtained the transgene-free mutant line after backcrossing, which was designated as *pct*, and used this mutant line in subsequent experiments.

### Mutation in *pct* drastically induced precocious vascular cambium formation in inflorescence stems

**Figure 1:**
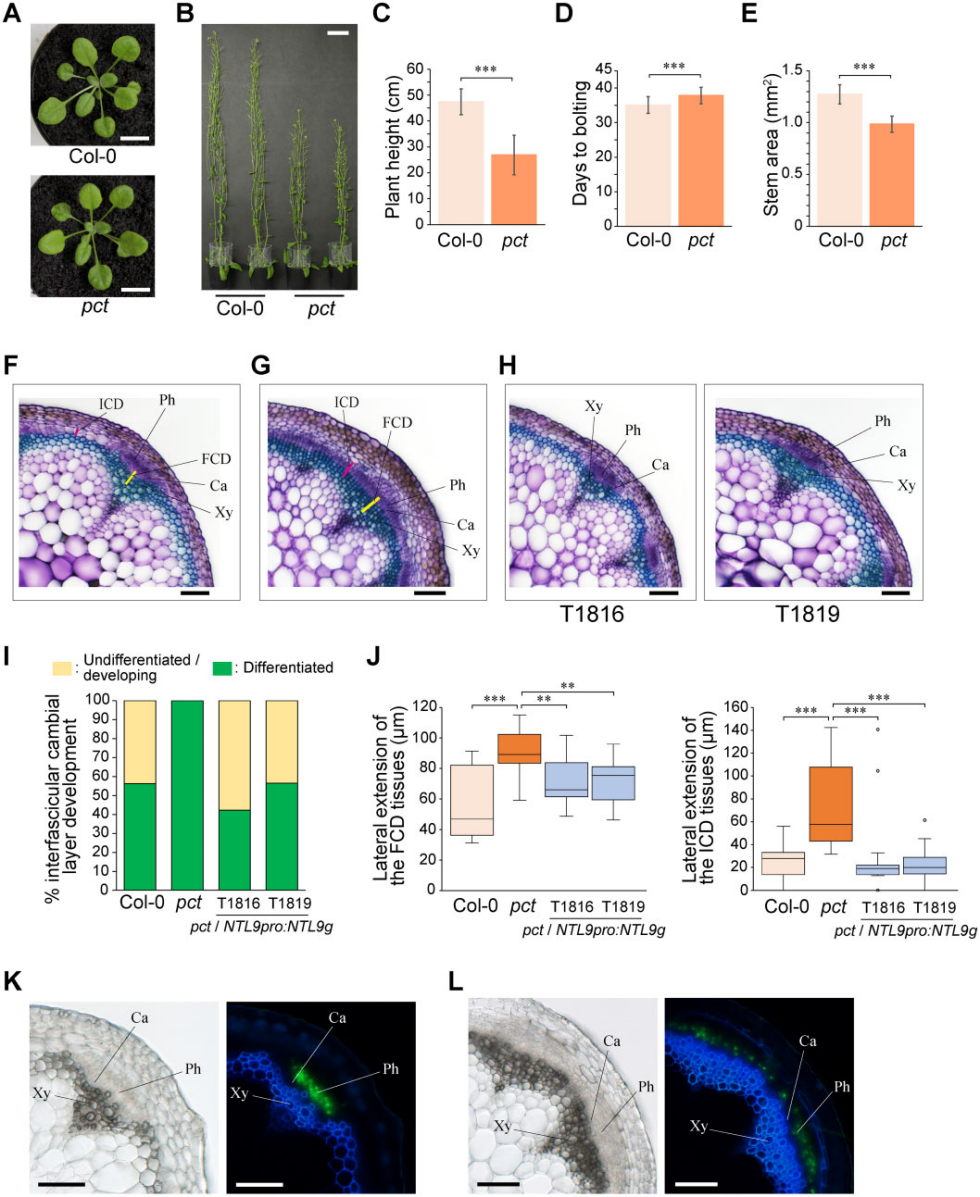
Arabidopsis *pct* mutant exhibited an increased secondary growth in inflorescence stems. A, Wild-type and *pct* mutant rosette plants (27-d-old). Scale bars, 10 mm. B, Wild-type and *pct* mutant plants (9-week-old). Scale bar, 50 mm. C, Height of 8-week-old plants. Values represent the mean ± SD. ***P <* *0.001; Student’s *t* test; *n *=* *16 for Col-0, *n *=* *20 for *pct*. D, Flowering time of wild-type and *pct* mutant. Flowering time was measured by counting the days when the primary shoot was 1-cm tall (1-cm bolting). Values represent the mean ± SD. ***P <* *0.001; Student’s *t* test; *n *=* *38 for Col-0, *n *=* *38 for *pct*. E, Stem area at 20 mm from the rosette base of primary inflorescence stems of 8-week-old plants. Values represent the mean ± SD. ***P <* *0.001; Student’s *t* test; *n *=* *5 for Col-0, *n *=* *6 for *pct*. F–H, Transverse sections of primary inflorescence stems from wild-type (F), *pct* mutant (G), and two independent *pct*/*NTL9pro:NTL9g* (T1816 and T1819) (H) plants. Basal regions (20 mm from the rosette base) of primary inflorescence stems in 8-week-old plants were used for observations. Enlarged images of vascular bundles are shown. FCD and ICD tissues, including the entire cambial cell layers, are shown by yellow and magenta bars, respectively. In the case of FCD tissues, the proximal and distal ends of cell files identified alongside the cambial cell layers within vascular bundles were defined as the inner and outer boundaries, respectively. In the case of ICD tissues, the starch sheath, which is a single layer of the innermost cells of the cortex, was defined as the outer boundary. The proximal end of the cell files identified alongside the cambial cell layers was defined as the inner boundary. Cross-sections were 100-μm thick and stained with toluidine blue. Ca, cambium; Ph, phloem; Xy, xylem. Scale bars, 100 μm. I, Differentiation levels of the basal regions of inflorescence stems. Differentiation levels were determined as a percentage of the number of differentiated interfascicular regions, based on whether the ICD tissues interconnected two adjacent primary vascular bundles, to total interfascicular regions analyzed. J, Quantitative analysis of cambial activity. The cambial activity was determined as a lateral extension of FCD and ICD tissues at basal regions (20 mm from the rosette base) of primary inflorescence stems of 8-week-old plants. Two independent *pct*/*NTL9pro:NTL9g* line plants (T1816 and T1819) were examined. See “Materials and methods” section for boxplot definition. **P <* *0.01; ***P <* *0.001; Kruskal–Wallis test followed by the Steel–Dwass test; *n *=* *16 for Col-0, *n *=* *17 for *pct*, *n *=* *26 for T1816, *n *=* *23 for T1819. K and L, *SUC2pro:GFP-RCI2a* reporter activity in the basal regions of the primary inflorescence stems from 8-week-old wild-type (K) and *pct* mutant (L) plants. Enlarged images of the vascular bundles with brightfield image (left panel) and UV lignin autofluorescence (blue) and GFP fluorescence (green; right panel) are shown. GFP fluorescence was restricted to phloem tissues within primary vascular bundles in wild-type inflorescence stems, whereas it was detected in both the primary phloem regions and distal tissues of interfascicular regions undergoing secondary growth in *pct* mutant inflorescence stems. Ca, cambium; Ph, phloem; Xy, xylem. Scale bars, 100 μm.

The *pct* mutant did not exhibit a whole-plant phenotype different from that of the wild-type, except for a slightly late-flowering phenotype and ∼40% reduction in stem height (Figure 1, A–D). In Arabidopsis inflorescence stems, secondary growth, such as the establishment of vascular cambium, is confined to the base of the stems, less than 10 mm above the uppermost rosette leaf (Little et al., 2002; Sehr et al., 2010). Under our growth conditions, a similar trend was found at 20 mm from the rosette base of 8- to 9-week-old wild-type inflorescence stems: seven to eight primary vascular bundles were observed that displayed few signs of secondary vascular growth although some interfascicular cambium formation could be seen (Figure 1F). Cross-sectional observation at corresponding tissues of the *pct* mutant revealed that the transverse area of the mutant inflorescence stem was slightly but significantly smaller than that of the wild-type (Figure 1E). However, a histologically defined area that had undergone an unusually high frequency of periclinal cell divisions as compared to the wild-type was visible in interfascicular regions of the *pct* mutant stems (Figure 1G). These differentiated interfascicular cambium-derived (ICD) tissues, including interfascicular cambium, extended laterally and encompassed adjacent fascicular cambium of primary vascular bundles, forming an almost continuous cylinder of meristematic cells comprising the vascular cambium (Figure 1, I and J). Accordingly, the *pct* mutant had larger arch-shaped vascular bundles, consisting of one or several connected bundles, although the spatial organization inside each vascular bundle was not disturbed in the *pct* mutant (Figure 1G).

To confirm whether the early established vascular cambium produced secondary phloem to the outside in the interfascicular regions of the *pct* mutant, we expressed membrane-anchored green fluorescent protein (GFP) (GFP fused to the N-terminus of RCI2a; Cutler et al., 2000) to prevent cell-to-cell movement under the phloem companion cell-specific sucrose-proton symporter 2 (*SUC2*) promoter (*SUC2pro:GFP-RCI2a*; Truernit and Sauer, 1995). Fluorescence microscopy analysis of the wild-type inflorescence stems showed that GFP fluorescence was only detected in phloem regions outside the fascicular cambium within primary vascular bundles and not in adjacent, interfascicular regions (Figure 1K). In contrast, in *pct* mutant inflorescence stems, we identified GFP fluorescence in the distal tissues of the interfascicular regions, which were present outside the interfascicular cambium, as well as primary phloem tissues (Figure 1L). This observation indicated that, in *pct* mutant stems, the secondary phloem was already differentiated in the interfascicular regions, indicating its early progression into the existing cylinder-like forms of phloem tissues.

Collectively, our results suggest that the recessive *pct* mutation induces the precocious formation of a ring-like domain of the vascular cambium following secondary phloem proliferation and that the *PCT* gene product negatively regulates secondary vascular growth progression in inflorescence stems of Arabidopsis.

### Mutation in *pct* enhanced cambial activity and early interfascicular cambium initiation in inflorescence stems

A developmental series of vascular tissues from the apex to the base of inflorescence stems in Arabidopsis has been reported (Altamura et al., 2001; Sehr et al., 2010). During secondary growth in stems, the vascular cambium develops through two developmental processes: lateral extension of fascicular cambium within vascular bundles and de novo formation of interfascicular cambium between interfascicular regions. It is therefore reasonable that the precocious development of vascular cambium-like structures observed in mutant inflorescence stems might be caused by increased activities of these cambia. To examine the influence of *pct* mutations on these cambial activities, we measured both the fascicular cambium-derived (FCD) and ICD tissues at the basal parts (20 mm above the rosette base), as a readout for their activities (Sehr et al., 2010; Brackmann et al., 2018; Figure 1, F, G, and J). The results revealed that the *pct* mutation increased FCD and ICD tissue production, indicating that the two cambial activities in the *pct* inflorescence stems were significantly enhanced.

**Figure 2:**
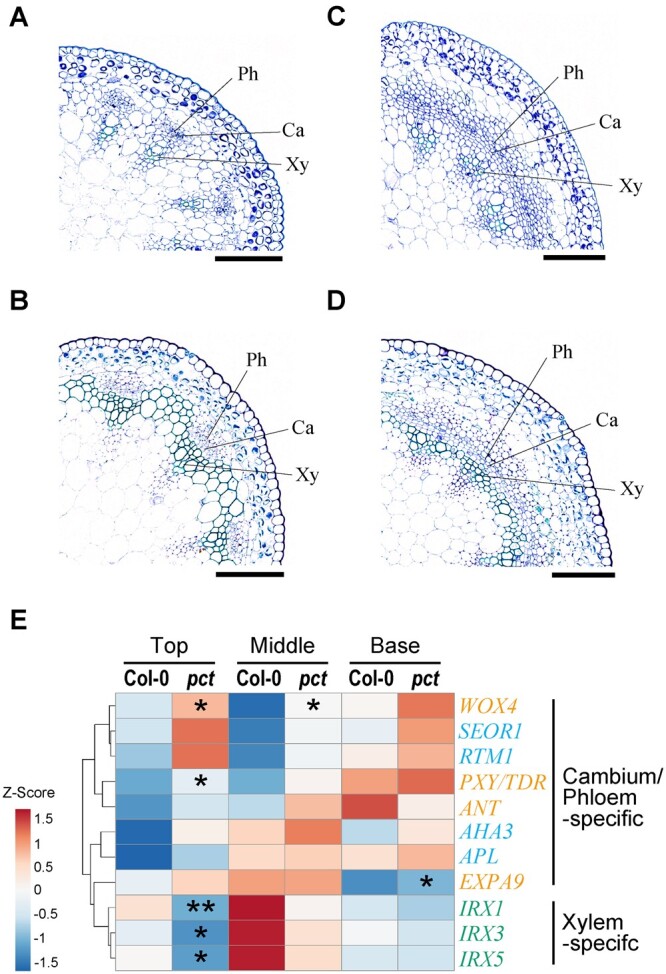
Mutation in *pct* caused an enhanced cambial activity at an early stage of stem vascular development. A–D, Transverse sections of the top regions (A and C) and middle regions (B and D) of primary inflorescence stems from 9-week-old wild-types (A and B) and *pct* mutants (C and D). Enlarged images of vascular bundles are shown. Cross-sections were 4-μm thick and stained with toluidine blue. Ca, cambium; Ph, phloem; Xy, xylem. Scale bars, 100 μm. E, Heatmap analysis of expression profiles of cambium-, phloem-, and xylem-marker genes for comparison of wild-type and *pct* mutant stems. Cambium-, phloem-, and xylem-marker genes are shown in orange, sky blue, and bluish green, respectively. Total RNA was isolated from three different regions (top, middle, and base) of 9-week-old inflorescence stems and subjected to RT-qPCR. The expression ratio of each gene to the *UBC9* gene was calculated for each sample. The values of samples for the top regions of the wild-type were set at 1 and used to determine the relative abundance for the other samples (raw data shown in Supplemental Figure S2). The clustering heatmap was generated using ClustVis (Metsalu and Vilo, 2015). Asterisks in the *pct* column indicate significant differences between the corresponding tissues of wild-type and *pct* mutant (*n *=* *3; *P <* *0.05; **P <* *0.01; Welch’s *t* test).

To further investigate the *PCT*-dependent regulation of cambial development during stem growth, we observed cross-sections obtained from primary inflorescence stems of 9-week-old wild-type and *pct* mutant plants in two sections, including the top region (10 mm below the shoot apex) and the middle region (a third of the way down). Histological observations revealed that the primary characteristics of the stem vasculature were visible in the top and middle regions of the wild-type stems: collateral vascular bundles were separated by interfascicular parenchyma, which is characterized by nonpericlinal divided cells (Figure 2, A and B). In contrast, an enlargement of vascular bundles and a precocious differentiation of interfascicular cambium were already evident at the top regions in the *pct* mutant stem although the spatial organization inside each vascular bundle was not disturbed, suggesting that the *pct* mutation induced abnormal stimulation of cambial activity at an early stage of stem growth (Figure 2C). Similarly, in the middle regions of the mutant stem, an almost continuous ring of vascular cambium with increased phloem tissues was observed along the circumference of the stem (Figure 2D), similar to that in the mutant basal regions.

The histological differences between the wild-type and *pct* mutant inflorescence stems led us to assess the expression levels of genes categorized as cambium-, phloem-, and/or xylem-specific using reverse transcription-quantitative polymerase chain reaction (RT-qPCR). These genes are the cambium markers *PHLOEM INTERCALATED WITH XYLEM*/*TRACHEARY ELEMENT DIFFERENTIATION INHIBITORY FACTOR RECEPTOR* (*PXY/TDR*; Fisher and Turner, 2007; Etchells and Turner, 2010), *WUSCHEL-RELATED HOMEOBOX4* (*WOX4*; Suer et al., 2011), and *AINTEGUMENTA* (*ANT*; Schrader et al., 2004; Randall et al., 2015); the cambium/xylem marker *α-EXPANSIN9* (*EXPA9*; Gray-Mitsumune et al., 2004); the phloem markers *SIEVE-ELEMENT-OCCLUSION-RELATED 1* (*SEOR1*; Froelich et al., 2011), *ALTERED PHLOEM DEVELOPMENT* (*APL*; Bonke et al., 2003), *RESTRICTED TEV MOVEMENT 1* (*RTM1*; Chisholm et al., 2001), and *ARABIDOPSIS H^+^-ATPASE ISOFORM 3* (*AHA3*; DeWitt and Sussman, 1995); and the xylem markers *IRREGULAR XYLEMs 1/3/5* (*IRX1*/3/5; Taylor et al., 2003). The clustering heatmap analysis for the marker genes showed clear separation between the wild-type and *pct* mutant stems (Figure 2E). In *pct* inflorescence stems, transcripts of genes classified as cambium- and/or phloem-related accumulated to higher levels even at the top regions, compared with those in the wild-type. Such a trend was also observed during whole-stem growth, supporting an earlier onset of cambial activity in mutant stems. In contrast, xylem-specific gene expression was reduced in the *pct* mutants (Figure 2E; Supplemental Figure S2).

**Figure 3:**
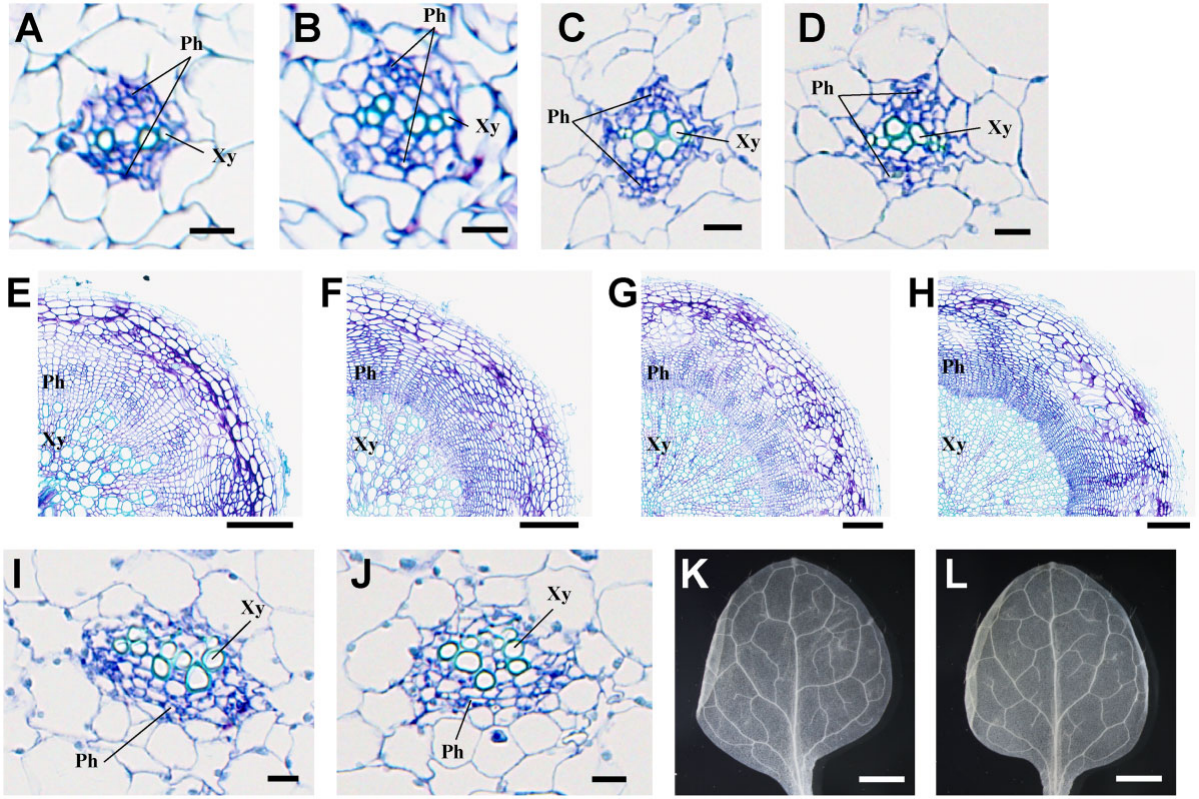
Vascular structure of roots, hypocotyls, and rosette leaves of the *pct* mutant. A–J, Transverse sections of roots, hypocotyls, and rosette leaves, from wild-type (A, C, E, G, and I), and *pct* mutant (B, D, F, H, and J) plants. The 7-d-old roots (A and B) and hypocotyls (C and D). The 9-week-old roots (E and F) and hypocotyls (G and H). Vasculature in the basal part of rosette leaves (I and J). Cross-sections were 4-μm-thick and stained with toluidine blue. Ph, phloem; Xy, xylem. K and L, Vein patterning in the first true leaves of 18-d-old wild-type (K) and *pct* mutant (L) plants. Scale bars, 10 μm (A–D, I, and J); 100 μm (E–H); 1 mm (K and L).

To further examine the effect of *pct* mutation on vascular organization throughout plant development, we observed the vascular tissues of roots, hypocotyls, and leaf veins of *pct* mutants. In contrast to inflorescence stems, 7-d-old *pct* mutant roots and hypocotyls did not display any obvious defects in the vasculature (Figure 3, A–D). A similar trend was observed in the roots and hypocotyls of 9-week-old wild-type and *pct* mutant plants, although the *pct* mutant appeared to exhibit a slightly smaller xylem area with a rough boundary and somewhat enlarged cambium and secondary phloem regions in the vasculature of the tissues (Figure 3, E–H). Moreover, vascular tissues and vein patterning of mutant leaves had the same organization as the wild-type (Figure 3, I–L). These observations suggest that the precocious differentiation in the vascular organization and the patterning caused by the *pct* mutation might be largely confined to inflorescence stems rather than throughout the plant body.

### *PCT* gene encodes NTL9 expressed during inflorescence stem vascular development

**Figure 4:**
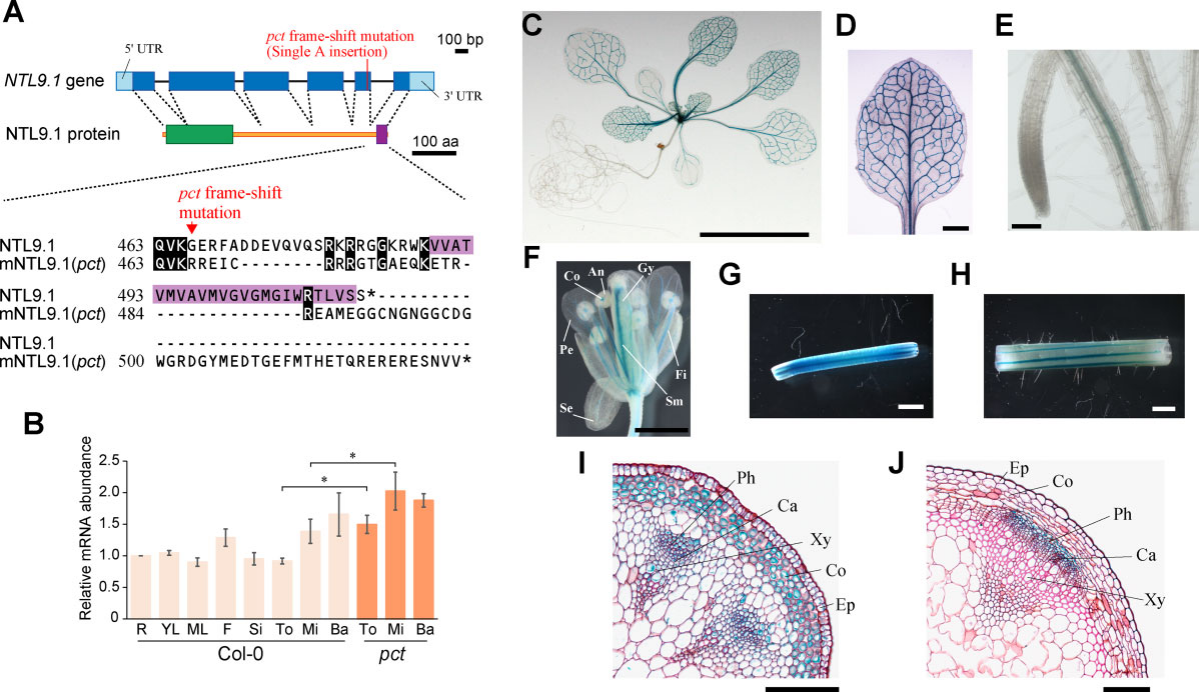
Molecular cloning and expression analysis of the *NTL9* gene. A, Structure of the *NTL9* gene and protein. The *NTL9* gene encodes three splice variants (*NTL9.1–3*; Supplemental Figure S5). Of these splice variants, *NTL9.1* is predominantly expressed as described in “Results” section. The upper part shows the *NTL9.1* gene structure, with boxes representing exons and thin lines representing introns. The *NTL9.1* open reading frame is depicted as blue boxes. The position of a single adenine nucleotide insertion in the *pct* mutant is indicated. The central part shows the NTL9.1 protein structure. The NAC transcriptional domain (9–159 aa) and TM domain (489–511 aa) are depicted as green and purple boxes, respectively. Functional domains were identified using the programs PROSITE (Falquet et al., 2002) and TMHMM (Krogh et al., 2001). The bottom part shows alignments of the C-terminal sequences of NTL9. In the mutated NTL9.1 resulting from *pct* mutation (mNTL9.1), a single adenine nucleotide insertion may be expected to result in a mistranslated sequence of the final 47 amino acids of NTL9.1, which might be replaced with 63 newly translated amino acids. The corresponding residues in the TM domain of NTL9.1 are highlighted in purple. Pairwise sequence alignment was obtained with NEEDLE from EMBOSS v6.6.0 (Rice et al., 2000). Identical residues are colored in black. Amino acid numbers for each protein are shown on the left. The asterisks represent stop codons. B, Expression of *NTL9* transcripts. Total RNA was isolated from different tissues of wild-type and *pct* mutant plants and subjected to RT-qPCR. Primers (F1 and R1), which span exons 3–4 of the *NTL9* gene (Supplemental Figure S5), were used. The expression ratio of each gene to the *UBC9* gene was calculated for each sample. The values of samples for root tissues of wild-type were set at 1 and used to determine the relative abundance for the other samples. R, 21-d-old roots; YL, 21-d-old third and fourth rosette leaves; ML, 21-d-old first and second rosette leaves; F, flowers; Si, siliques; To, top regions of 9-week-old inflorescence stems; Mi, middle regions of 9-week-old inflorescence stems; Ba, basal regions of 9-week-old inflorescence stems. Values represent the mean ± SD. of three biological and technical replicates (*P <* *0.05; Welch’s *t* test). C–J, Histochemical analysis of GUS reporter activity in Arabidopsis *NTL9pro:GUS* transgenic plants. An 18-d-old plant (C). The third rosette leaf (D). The roots (E). The flowers (F). An, anther; Co, connective; Fi, filament, Gy, gynoecium; Pe, petal; Se, sepal; Sm, Septum. G and H, Approximately 1.0-cm inflorescence stem segments as positioned 10 mm below the shoot apex (G) and 20 mm above the uppermost rosette leaf (H) of 9-week-old transgenic plants. I and J, Transverse sections of the top (I) and basal (J) regions of primary inflorescence stems from 9-week-old transgenic plants. Enlarged images of vascular bundles are shown. After GUS staining, stem tissues were embedded in paraffin. Cross-sections were 10-μm-thick and counterstained with safranin O. Ca, cambium; Co, cortex; Ep, epidermis; Ph, phloem; Xy, xylem. Scale bars, 10 mm (C); 1 mm (D, F–H); 100 μm (E, I, and J).

Using a map-based approach, we found that one base insertion (a nucleotide A), which caused a single frameshift event, occurred in the coding region of *NTL9* (*At4G35580*; Figure 4A; Supplemental Figure S3). To determine whether *NTL9* represents the *PCT* gene, we generated transgenic *pct* mutants carrying the wild-type *NTL9* gene under the transcriptional control of either its native promoter or the cauliflower mosaic virus (*CaMV*) *35S* promoter (*pct*/*NTL9pro:NTL9g* and *pct*/*35Spro:NTL9g*, respectively). Cross-sectional observational data from primary inflorescence stems showed that the stem phenotype of these transgenic plants was restored to that of the wild-type plants, with the primary vascular bundles clearly separated by interfascicular parenchyma and thus exhibited few signs of secondary vascular growth—typical of wild-type inflorescence stems (Figure 1, H–J; Supplemental Figure S4). The results indicated that the *NTL9* gene was indeed the *PCT* gene, and the promoter region we used was at least functional in the stem.

It has been reported that the expression of *NTL9* is activated in response to osmotic stress and senescence (Yoon et al., 2008). However, few studies have focused on the developmental and spatial expression patterns of the *NTL9* gene, except for its expression in the vasculature of cotyledons (Kim et al., 2007; Yoon et al., 2008). Therefore, we examined the expression profile of the *NTL9* gene using RT-qPCR analysis (Figure 4B; Supplemental Figure S5). *NTL9* had higher levels of expression in the inflorescence stem, especially its basal region, although it was ubiquitously expressed in all plant tissues.

To further determine the tissue- or cell-type-specific expression patterns of the *NTL9* gene, we generated transgenic Arabidopsis plants expressing the β-glucuronidase (GUS) reporter gene under the transcriptional control of the *NTL9* promoter (*NTL9pro:GUS*). In most transgenic plants, vascular-specific GUS staining was detected in aerial tissues such as the cotyledons and rosette leaves, whereas very weak staining was observed in the vascular tissues of root tissues, along with the sepals and petals of mature flowers (Figure 4, C–F).

Although *NTL9* expression has been detected in various cell types within inflorescence stems, higher *NTL9* expression levels were detected in the cambium/phloem domains than in the pith/xylem domains using phloem companion cell-specific or fluorescence-activated nucleus sorting– and laser capture microdissection–derived transcriptome analyses (Supplemental Figure S6; You et al., 2019; Shi et al., 2021). We also observed GUS staining patterns of the top and basal regions of primary inflorescence stems from 9-week-old *NTL9pro:GUS* transgenic plants. Strong GUS activity, especially in the vascular tissues, was observed in both the top and basal regions (Figure 4, G and H). Cross-sectional observation revealed that, in the top regions of the stem, GUS activity was detected in every cell type within the primary vascular bundles as well as the entire cortex and epidermis (Figure 4I). However, in the stem basal regions, the *NTL9* promoter was mostly active in the distal domain (phloem side) of the cambium and differentiated phloem tissues, including the developing phloem cells and phloem parenchyma within vascular bundles (Figure 4J). Overall, *NTL9* is transcriptionally regulated in cell type- and developmental stage-specific manners along the whole-stem vasculature and represses developmental transition to the secondary growth phase.

### Precocious secondary growth in stems of *pct* mutants is caused by a disruption of the TM domain of NTL9.1

Three unique splice variants of *NTL9* (*NTL9.1*, *NTL9.2*, and *NTL9.3*) were identified (Supplemental Figure S5; Block *et al.*, 2014). The *NTL9.1* variant consists of six exons and encodes a 512-amino acid protein composed of the NAC transcriptional domain in its N-terminal region, and an α-helical TM domain located in its distant C-terminal region (Figure 4A). Both *NTL9.2* and *NTL9.3* have been reported to use an alternative acceptor site within exon 6, which leads to the encoding of the NAC transcription factor without the C-terminal TM domain (Supplemental Figure S5; Block et al., 2014). NTL9.2 and NTL9.3 proteins were identical except for one lysine residue insertion (at amino acid position 474) of NTL9.2. A frameshift *pct* mutation on the *NTL9.1* splice variant resulted in a mistranslated sequence of the final 47 amino acids of the NTL9.1 protein that might be replaced with newly translated 63 amino acids, which were identical to the C-terminal 55-amino acid sequence of the NTL9.2/NTL9.3 proteins. Therefore, the mutated NTL9.1 protein as well as the NTL9.2/NTL9.3 proteins lack the TM domain (Supplemental Figure S5).

**Figure 5:**
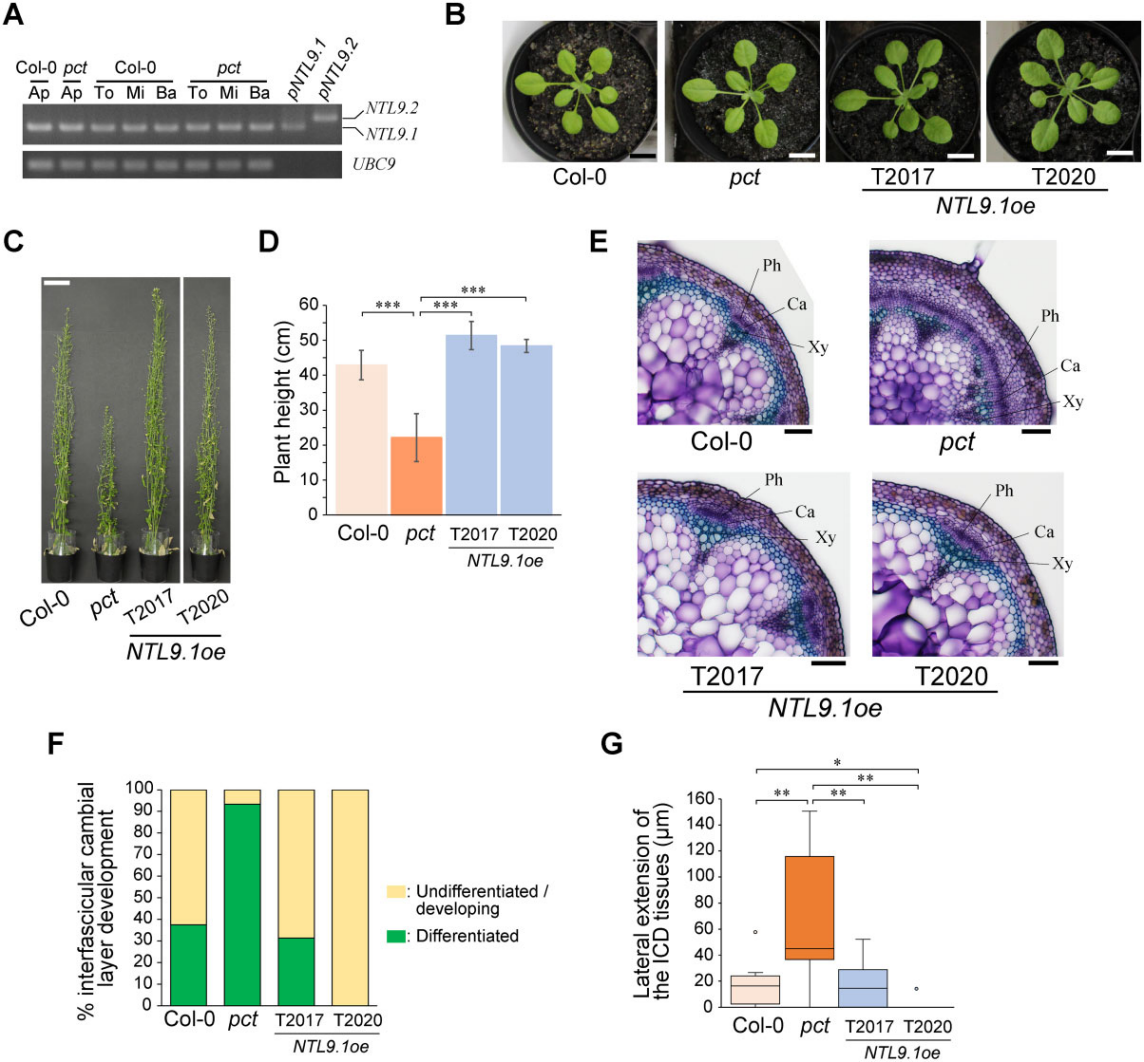
The major splice variant of *NTL9*, *NTL9.1*, negatively regulates the transition from primary to secondary growth in Arabidopsis inflorescence stems. A, Expression of *NTL9* splice variants transcripts in wild-type and *pct* mutant plants. Total RNA was isolated from different tissues of wild-type and *pct* mutant plants and subjected to RT-PCR. Primers (F2 and R2), which span exon 5–6 of the *NTL9* gene (shown in Supplemental Figure S5), were used to distinguish between *NTL9.1* and *NTL9.2*/*NTL9.3* splice variants. Ap, aerial parts of 21-d-old plants; To, top regions of 9-week-old inflorescence stems; Mi, middle regions of 9-week-old inflorescence stems; Ba, basal regions of 9-week-old inflorescence stems; *pNTL9.1*, plasmid control of *NTL9.1* splice variant; *pNTL9.2*, plasmid control of *NTL9.2* splice variant. B and C, The 25-d-old (B) and 9-week-old (C) *NTL9.1oe* line plants. Two independent *NTL9.1oe* line plants (T2017 and T2020) were examined. Scale bars, 10 mm (B); 50 mm (C). D, Height of 9-week-old plants. Two independent *NTL9.1oe* line plants (T2017 and T2020) were examined. Values represent the mean ± SD. ***P <* *0.001; one-way ANOVA followed by the Tukey–Kramer test; *n *=* *4 for Col-0, *n *=* *4 for *pct*, *n *=* *3 for T2017, *n *=* *4 for T2020. E, Transverse primary inflorescence stem sections from *NTL9.1oe* line plants. Basal parts (20 mm from the rosette base) of primary inflorescence stems in 9-week-old plants were used for observations. Two independent *NTL9.1oe* line plants (T2017 and T2020) were examined. Enlarged images of vascular bundles are shown. Cross-sections were 100-μm thick and stained with toluidine blue. Ca, cambium; Ph, phloem; Xy, xylem. Scale bars, 100 μm. F, Differentiation levels of the basal regions of inflorescence stems. Differentiation levels were determined as a percentage of the number of differentiated interfascicular regions, based on whether the ICD tissues interconnected two adjacent primary vascular bundles, to total interfascicular regions analyzed. Two independent *NTL9.1oe* line plants (T2017 and T2020) were examined. G, Quantitative analysis of cambial activity. The cambial activity was determined as a lateral extension of ICD tissues at basal regions (20 mm from the rosette base) of primary inflorescence stems of 9-week-old plants. See Figure 1, F and G for the definition of ICD tissues. Two independent *NTL9.1oe* line plants (T2017 and T2020) were examined. See “Materials and methods” section for boxplot definition. *P <* *0.05; **P <* *0.01; Kruskal–Wallis test followed by the Steel–Dwass test; *n *=* *16 for Col-0, *n *=* *15 for *pct*, *n *=* *7 for T2017, *n *=* *8 for T2020.

We conducted reverse transcription PCR (RT-PCR) to investigate which splice variants of *NTL9* are expressed in *Arabidopsis*. The results showed that the *NTL9.1* splice variant was the most prevalent form during whole-plant development (Figure 5A), raising the possibility that the recessive *pct* mutant stem phenotype was caused by functional disruption of the dominant form of NTL9, namely, NTL9.1.

The properties of the *NTL9* splice variants led us to explore whether *NTL9.1* could suppress early interfascicular cambium initiation observed in the *pct* mutant inflorescence stems. To this end, we generated transgenic *pct* mutant plants expressing the *NTL9.1* splicing variant under the transcriptional control of the *CaMV 35S* promoter (*NTL9.1oe*). The whole-plant phenotype of *NTL9.1oe* line plants was almost the same as that of the wild-type plants (Figure 5, B–D). Cross-sectional observations of primary inflorescence stems revealed that *NTL9.1oe* inflorescence stems had few signs of secondary vascular tissue; the primary vascular bundles were separated from each other by nonpericlinal divided interfascicular parenchyma cells and arranged in a ring, resulting in restoration of differentiation levels of the stem and cambial activity to levels similar to that of the wild-type (Figure 5, E–G). These results indicated that *NTL9.1* was sufficient to fully complement the precocious formation phenotype of vascular cambium caused by the *pct* mutation, consistent with the predominant *NTL9.1* expression among *NTL9* splice variants.

We further examined whether the *pct* mutant expressed endogenous mutated *NTL9* transcripts, and if so, whether the transcripts maintained a normal splicing event. RT-qPCR showed that endogenous mutated forms of *NTL9* transcripts accumulated to a greater degree in aerial parts and stem tissues of *pct* mutants than in wild-type (Figure 4B; Supplemental Figure S7), indicating that *NTL9* expression was fine-tuned by negative feedback control. Furthermore, RT-PCR and sequence analysis revealed that mutated *NTL9.1* transcripts were the predominant forms of mutated transcripts and were normally spliced (Figure 5A; Supplemental Figure S5). Considering that mutated NTL9.1 protein displayed high similarity to NTL9.2/NTL9.3 proteins, which possess an NAC transcriptional domain but lacked a TM domain at the C-terminus (Supplemental Figure S5), these results suggest that the TM domain of NTL9.1 is required for inherent NTL9 as a negative regulator of the onset and progression of the secondary growth phase in inflorescence stems.

### Transcriptional analysis of genes involved in the secondary growth of *pct* mutant inflorescence stems

**Figure 6:**
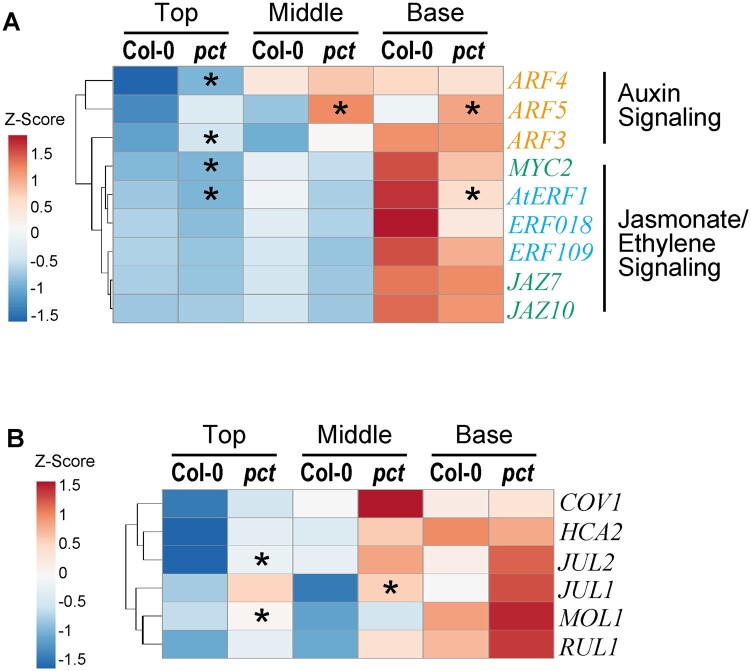
Transcriptional profile of *pct* mutants during inflorescence stem growth. A, Heatmap analysis of the expression profiles of phytohormone-regulated genes for the comparison of wild-type and *pct* mutant stems. Auxin, ethylene, and jasmonate-regulated genes are shown in orange, sky blue, and bluish green, respectively. B, Heatmap analysis of the expression profiles of stem secondary growth-associated genes for the comparison of wild-type and *pct* mutant stems. Total RNA was isolated from three different regions (top, middle, and base) of 9-week-old inflorescence stems and subjected to RT-qPCR. The expression ratio of each gene to the *UBC9* gene was calculated for each sample. The values of samples for the top regions of the wild-type were set at 1 and used to determine the relative abundance for the other samples (raw data shown in Supplemental Figure S8). The clustering heatmap was generated using ClustVis (Metsalu and Vilo, 2015). Asterisks in *pct* column indicate significant differences between the corresponding tissues of wild-type and *pct* mutant (*n *=* *3; *P <* *0.05; Welch’s *t* test).

Phytohormones, especially auxin, ethylene, and jasmonate, have been shown to induce differentiation of interfascicular cambium during the early stage of stem secondary growth (Sehr *et al.*, 2010; Agusti *et al.*, 2011; Etchells *et al.*, 2012; Mazur *et al.*, 2014; Brackmann *et al.*, 2018). To assess if the *pct* mutation stimulates the secondary growth of inflorescence stems in a phytohormone-regulated manner, we compared the expression of genes involved in these signaling pathways. These genes included the auxin-regulated *AUXIN RESPONSE FACTORs* (*ARF3–ARF5*; Brackmann et al., 2018; Liu et al., 2018), the jasmonate-regulated *JASMONATE-ZIM-DOMAINs* (*JAZ7* and *JAZ10*) and *MYC-transcription factor 2* (*MYC2*; Sehr et al., 2010; Yu et al., 2016), and the ethylene-regulated *ETHYLENE RESPONSE FACTORs* (*AtERF1*, *ERF018*, and *ERF109*; Etchells *et* al., 2012). RT-qPCR analysis revealed that *ARF3–5* and *WOX4*, whose transcription is also induced by auxin (Suer et al., 2011), were already transcriptionally upregulated during the early stage of mutant inflorescence stem development (Figures 2E and 6A; Supplemental Figures S2A and S8A). In contrast, reduced levels of transcripts of any of the analyzed genes involved in signal responses for ethylene (*AtERF1*, *ERF018*, and *ERF109*) and jasmonate (*JAZ7*, *JAZ10*, and *MYC2*) were observed in the whole-mutant inflorescence stems, though these gene expression levels were drastically higher in the basal regions than in the top regions (Figure 6A; Supplemental Figure S8A). Thus, the increased secondary growth observed in *pct* mutant inflorescence stems might have resulted from increased auxin signaling, but not jasmonate and ethylene signaling.

To further determine the indications for the genetic network of *NTL9* during vascular development in stems, we analyzed the expression of genes that function as positive or negative regulators of vascular cambium formation or phloem cell differentiation in *pct* mutants. These genes included *COV1* (Parker *et al.*, 2003), *HCA2* (Guo *et al.*, 2009; Miyashima *et al.*, 2019), *MOL1* and *RUL1* (Agusti *et al.*, 2011; Gursanscky *et al.*, 2016), as well as *JULGI*s *1/2* (*JUL1/2*, Cho *et al.*, 2018). The results showed that, in the top and middle regions of inflorescence stems of the *pct* mutant, all the investigated gene transcripts accumulated to a greater extent than the wild-type (Figure 6B; Supplemental Figure S8B), indicating that the complex regulatory control of vascular cambium development mediated by *NTL9* along with these regulatory genes ensures fine-tuned secondary vascular development.

## Discussion

In this study, we identified NTL9 as a negative regulator of vascular cambium development during secondary growth in stems. The Arabidopsis recessive *pct* mutant exhibited enhanced vascular cambium formation in the circular form, followed by increased secondary phloem production. Cross-sectional analysis revealed that the *pct* mutation induced precocious differentiation of interfascicular cambium during the early stage of stem development. Furthermore, the expression of cambium/phloem-marker genes was elevated during *pct* mutant stem development. Map-based cloning revealed that the *PCT* gene encoded NTL9 and the *pct* mutation caused a frameshift in the C-terminus, resulting in a disruption of the TM domain of NTL9. Of the three *NTL9* splice variants (*NTL9.1–3*), *NTL9.1* is the predominant form and only NTL9.1 possesses the TM domain. These results suggest that NTL9 functions as a negative regulator of vascular cambium differentiation through precise regulation of its TM domain.

Monocotyledonous species usually lack secondary growth in stems. A position-specific iterated-BLAST search (Altschul et al., 1997) using NTL9 as a query against the NCBI-NR (nonredundant) protein database (as of December 1, 2021) revealed that all hits (*E*-value <2e^−89^; >34.98% identity) were found in dicotyledonous species, thus supporting its functional involvement in secondary vascular developmental processes. The *pct* mutant stem is characterized by enhanced vascular cambium formation through a precocious de novo establishment of interfascicular cambium. This characteristic was evident at the top regions of the mutant stem, where the *NTL9* gene was broadly expressed in the stelar tissues. This finding indicated that *NTL9* may directly suppress the establishment of the cambium identity, especially in interfascicular regions. However, for several reasons, such inference may seem implausible. First, a limited number of stem cell identities emerge based on the spatial separation of ligands and receptors in stem vasculature (Etchells and Turner, 2010; Miyashima et al., 2013; Nieminen et al., 2015; Etchells et al., 2016). PXY/TDR, a cambium-specific leucine-rich repeat receptor-like kinase, acts as a receptor of the peptide ligands CLE41/CLE44/TDIF, which are produced in the phloem and move toward the procambium. Through its interaction with CLE peptides, PXY stimulates WOX4 transcription factor activity and regulates the proliferation of cambial cells and vascular tissue patterning in stems. Consistently, ectopic *CLE41* expression and *pxy* mutation both cause misaligned cell divisions, and subsequently, organizational defects in vascular tissues (Fisher and Turner, 2007; Etchells and Turner, 2010). In contrast, the *pct* mutant did not cause organizational defects in stem vascular bundles as positional information and highly polarized periclinal divisions were maintained. Second, some studies have suggested that the activation of fascicular cambium induces interfascicular cambium formation (Little et al., 2002; Sehr et al., 2010; Mazur et al., 2014) and that both *PXY* and *WOX4* are required for cambium initiation of the interfascicular and fascicular regions because low interfascicular cambium formation was observed in these null mutants (Agusti et al., 2011; Suer et al., 2011). In the present study, we observed enhanced cambial activity not only in the interfascicular regions but also in the fascicular region of the *pct* mutant. Accordingly, the expression of genes categorized as cambium-related, including *PXY* and *WOX4*, was enhanced during mutant stem development, especially in the top regions compared to the wild-type, reflecting the increased cambial activity in the *pct* mutant stem. Such observations are also supported by our observation that phytohormone auxin signaling, which has been shown to induce cambial activity (Agusti et al., 2011; Suer et al., 2011; Mazur et al., 2014; Brackmann et al., 2018), was stimulated during *pct* mutant development. Finally, secondary growth in wild-type stems was confined to the basal region close to the rosette base. In this region, *NTL9* was predominantly expressed in the phloem domain within vascular bundles, apart from the domain of interfascicular cambium establishment. Therefore, we conclude that the primary function of *NTL9* is to suppress the developmental transition to secondary growth by negatively regulating fascicular, as well as interfascicular, cambial activity, but not by establishing cambium identity.

MTFs are transcription factors with TM domains under unique regulation to ensure prompt transcriptional responses to intracellular and environmental stimuli (Chen et al., 2008; Seo et al., 2008; Slabaugh and Brandizzi, 2011; Seo, 2014). First, MTFs are stored in dormant forms in association with parent membranes, such as the plasma membrane or endoplasmic reticulum membrane. Upon internal or external stimuli, MTFs are released from the parent membranes and relocated to the nucleus, where they modulate the transcription of target genes. Although its subcellular localization remains controversial, full-length NTL9 appears to be mainly attached to the endoplasmic reticulum membrane (Kim et al., 2007; Yoon et al., 2008; Block et al., 2014; Liang et al., 2015; Zheng et al., 2015; Guo et al., 2017). Upon osmotic stress, NTL9 is liberated from the membrane, enters the nucleus, and subsequently induces the expression of a subset of *SAGs* (Yoon et al., 2008).

The dominant form of NTL9, NTL9-D, which lacks 182 residues of the C-terminal region, including the TM domain, and is equivalent to the released NTL9.1, is transcriptionally active and is transported to the nucleus (Yoon et al., 2008). Overexpression of *NTL9-D* in Arabidopsis induces a dwarf phenotype with small, curved, and occasionally pale-green leaves (Yoon et al., 2008). Therefore, it was notable that the *pct* mutation conferred a recessive phenotype because this frameshift mutation disrupts the TM domain of NTL9.1, allowing the mutated NTL9.1 to be released from the membrane. Furthermore, in contrast to *NTL9-D* overexpressors, transgenic plants overexpressing the full-size *NTL9* exhibited a whole-plant phenotype like that of the wild-type. Such phenotypic discrepancy may be due to the difference in functional activities among the different forms of released NTL9. This inference may be supported by reports of other NTLs, such as NTM1/NTL12, NTM2, and NTL6 (Kim et al., 2006; Seo et al., 2010a; Park et al., 2011). *ntm1-D* is the dominant mutant of *NTM1*, which encodes a 473 amino acid protein, and presents a serrated leaves phenotype (Kim et al., 2006). The mutation is caused by T-DNA insertion in the C-terminal region of NTM1, which is equivalent to expressing the truncated form of NTM1 containing residues 1–327 (NTM1ΔC). NTM1ΔC and another truncated form of NTM1 (NTM1ΔTM), which contains the residues 1–445 and lacks only the TM domain at the distant C-terminal end, are both transported to the nucleus. However, overexpression of NTM1ΔC, but not that of full-length NTM1 or NTM1ΔTM, causes Arabidopsis plants to exhibit serrated leaves. This finding highlights the potential inhibitory activity in the C-terminal region, which is present in NTM1ΔTM but not in NTM1ΔC. Similarly, analysis of their transcriptional activation using a series of truncated forms of NTM2 and NTL6 has provided evidence that the release forms of the NTLs are not sufficient for their transcriptional activity, and the intermediate regions between NAC and TM domains have an inhibitory role in their activity (Kim et al., 2007; Seo et al., 2010a; Park et al., 2011). The upstream region from the TM domain in NTL9, therefore, could exhibit potential inhibitory activity against transcription, also indicating that the precise excision of the C-terminal region should be responsible for not only the release from the membrane but also the transcriptional activation of NTL9. Notably, unlike the case for *NTL9-D*, overexpression of full-size *NTL9* did not induce any visible phenotypic effects. This indicated that the membrane-releasing process itself is the primary regulatory step for NTL9 activation because the proteolytic process of NTL9 is irreversible.

Converging evidence indicates that NTLs provide adaptive strategies for plants to survive fluctuating environmental conditions (Chen et al., 2008; Slabaugh and Brandizzi, 2011; Seo, 2014; Meng et al., 2019). For example, NTL6 coordinates cold stress tolerance and pathogen resistance responses (Kim et al., 2007; Seo et al., 2010a), whereas NTL7 is involved in transcriptional regulation of the mitochondrial stress response, autophagy, cell death, and senescence (Meng et al., 2019). Similarly, NTL9 regulates multiple cellular signaling pathways, including osmotic stress signaling, developmental leaf senescence, and innate immune response (Yoon et al., 2008; Block et al., 2014; Zheng et al., 2015; Guo et al., 2017). Depending on whether they are under nonstress or biotic or abiotic stress conditions, plants assign their available energy toward either growth or defense through a complicated hormone crosstalk to optimize their fitness and survival. This phenomenon is known as the growth-defense trade-off concept (Cortleven et al., 2019). For example, the phytohormone SA induced by biotic stress activates immune responses such as pathogenesis-related gene expression and simultaneously interferes with auxin transport and signaling, thereby repressing growth-related responses in plants (Wang et al., 2007; Du et al., 2013; Huot et al., 2014; van Butselaar and Van den Ackerveken, 2020; Zhong et al., 2021). *NTL9* is essential for the transcriptional induction of SA biosynthesis- and defense-related genes such as *ICS1* and *PR1* (Zheng et al., 2015; Guo et al., 2017). Moreover, NTL9 functions as a highly connected cellular hub within the immune system network and is therefore targeted by effectors from pathogens to facilitate pathogen fitness (Mukhtar et al., 2011). Therefore, *NTL9* may play a key role in fine-tuning the balance of growth and defense states in stem secondary growth, which could be supported by the observation that auxin-regulated *ARFs* were transcriptionally activated in the *pct* stem.

Notably, the proteolytic processing of NTL6, which is structurally closest to NTL9, is induced by cold-induced changes in membrane fluidity (Kim et al., 2007; Seo et al., 2010b). Similarly, osmotic stress has been shown to induce proteolytic processing of NTL9 (Yoon et al., 2008). To enhance our understanding of NTL9 function in stem secondary growth, more insights are required into its release from the membrane, the direct transcriptional targets of NTL9, and the up- and down-stream factors of NTL9. Considering the unique hub function of NTL9 between various signaling pathways, we envisage exceptionally intricate cambium regulation during secondary growth. Furthermore, manipulating NTL9 as a critical regulator of vascular cambium ontogenesis would help meet the increasing demand for environmentally friendly and economically viable renewable resources for sustainable development.

## Materials and methods

### Plant materials and growth conditions

The Arabidopsis (*A. thaliana*) wild-type, *phloem circular-timing* (*pct*) mutant, and transgenic plants used in the present study were of the Columbia (Col-0) ecotype. The original *pct* mutant, *pct.o* (*phloem circular-timing. original*), was unexpectedly identified from T-DNA (*pPXYpro:AtPP2CF1*; Supplemental Method S1 with the primers listed in Supplemental Table S2)-tagged T2 populations; however, the *pct* mutation was not linked with the T-DNA insertion, as described in “Results” section. The *pct.o* mutant was first back-crossed twice with wild-type Col-0 to remove T-DNA and self-pollinated to give rise to the *pct* mutant that is used in this study. The *pct* mutant demonstrated the absence of T-DNA based on PCR analysis using primers listed in Supplemental Table S3.

Seeds were sown aseptically on Murashige and Skoog (MS) solid medium (0.5% [w/v] gellan gum) supplemented with 1% (w/v) sucrose or on soil Supermix A (Sakata, Kanagawa, Japan). After stratification for 3 d at 4°C, plants were grown in a controlled growth chamber (at 22°C and ∼60% relative humidity) under long-day conditions (16 h:8 h, light:dark, ∼50 μmol m^−2^ s^−1^ white fluorescent light). Seedlings of plants grown on solid medium were transferred to soil at 21 d after imbibition and fertilized weekly with Hyponex (1:1,000 dilution; Hyponex Japan, Osaka, Japan).

### High-resolution mapping of *PCT* locus and identification of *pct* mutation

Procedures for map-based cloning of the *PCT* gene are provided in Supplemental Method S2 with the primers listed in Supplemental Table S4. After identification of the mutated site, the *pct* mutation was genotyped using a derived cleaved amplified polymorphic sequence marker (Supplemental Table S3).

### Construction of plasmids and transgenic plants

The floral dip method was used for Arabidopsis transformation (Clough and Bent, 1998). Procedures for the construction of plasmids are provided in Supplemental Method S1, with the primers listed in Supplemental Table S2.

### Histological analysis of inflorescence stems

Underneath the cauline branches, the expanding vascular bundles consisting of two to three bundles were often observed. To avoid this asymmetric effect of cauline branches on tissue patterning, only Arabidopsis plants, in which the first internode was at least 30-mm long, were selected for the observation of the basal region (20 mm from the rosette base) of the primary inflorescence stems (Little et al., 2002; Sehr et al., 2010). In addition, to avoid the similar effect in pedicels, the stem regions between the pedicels at the top region (10 mm below the shoot apex) and the middle region (a third of the way down) were selected for observation.

GUS staining was performed as previously described (Sugimoto et al., 2014), and the tissue samples were incubated overnight at 37°C. For transverse sections, tissue samples were embedded in agarose blocks and sectioned into 100-μm-thick slices using a micro-slicer (DTK-1000; Dosaka, Kyoto, Japan) or sectioned into 4- or 10-μm-thick slices after paraffin embedding (GenoStaff, Tokyo, Japan). For toluidine blue staining, tissue sections were incubated with 0.05% (w/v) toluidine blue for either 1 min (agarose-removed sections) or 15 min (de-paraffinized sections; GenoStaff). Safranin O staining was performed at GenoStaff. Briefly, the de-paraffinized sections were incubated with 0.5% (w/v) iron hematoxylin and washed. Subsequently, the tissue sections were placed in 0.02% (w/v) Fast Green FCF (in 1% (v/v) acetate acid) for 2 min, washed, and stained with 0.1% (w/v) safranin O for 3 min. For fluorescence microscopy analysis, agarose-embedded tissue samples were sectioned into 100-μm-thick slices and cleared using ClearSee solution (10% (w/v) xylitol, 15% (w/v) sodium deoxycholate, 25% (w/v) urea; Kurihara et al., 2015) for up to 1 week.

Optical and fluorescence images were obtained under a stereomicroscope (SZX12; Olympus, Tokyo, Japan), a BX51 fluorescence microscope (Olympus), a BZ-9000 Biorevo fluorescence microscope (Keyence, Osaka, Japan), or a NanoZoomer S210 (Hamamatsu Photonics, Shizuoka, Japan). Fluorescence excitation wavelengths were 470–490 nm and 330–385 nm for GFP and lignin, respectively. The emitted fluorescence was detected through 510–550 nm bandpass and >420 long-pass filters for GFP and lignin, respectively. The acquired images were processed and assembled using Adobe Photoshop (Adobe Systems, San Jose, CA) and ImageJ software (Schneider et al., 2012; http://rsb.info.nih.gov/ij/).

### RT-qPCR

Total RNA was isolated using an RNeasy Plant Mini Kit (Qiagen, Valencia, CA) with on-column DNase treatment (RNase-Free DNase Set; Qiagen, Hilden, Germany). The cDNA was synthesized using a High-Capacity RNA-to-cDNA Kit (Applied Biosystems, Foster City, CA), and subjected to RT-qPCR amplification using the PowerUp SYBR Green Master Mix (Applied Biosystems) with gene-specific primers listed in Supplemental Table S5. Quantification was conducted using the QuantStudio 3 Real-Time PCR System (Thermo Fisher Scientific, Waltham, MA) and QuantStudio Design & Analysis v.1.4.1 (Thermo Fisher Scientific). The expression ratio of each gene to the *UBC9* gene was calculated for each sample. The values of samples for roots or top regions of inflorescence stems of wild-type plants were set at 1 and used to determine the relative abundance for the other samples. A clustering heatmap was generated using ClustVis (Metsalu and Vilo, 2015). The optional parameters were set as follows: no transformation, row-centering, unit variance scaling, and clustering using Euclidean distance and average linkage for rows.

### Rapid amplification of cDNA 3′ ends (3′-RACE) and RT-PCR

3′-RACE was performed using the 3′-Full RACE core set (Takara, Shiga, Japan) according to the manufacturer’s instructions. The *NTL9*-specific primers are listed in Supplemental Table S6. The cDNA was also subjected to RT-PCR amplification, with *NTL9*-specific primers listed in Supplemental Table S5.

### Statistical analyses

All analyzed datasets were assessed for normal distribution using the Shapiro–Wilk test and for variance homogeneity using Levene’s test. Significant differences between two datasets were assessed using the Student’s *t* test or Welch’s *t* test, based on variance homogeneity. For multiple comparisons among three or more datasets, one-way ANOVA followed by the Tukey–Kramer test (for normal distribution) or the Kruskal–Wallis test followed by the Steel–Dwass test (for non-normal distribution) were performed.

### Boxplot

Boxplots were generated with standard boxplot settings: the median (central mark), the 25th and 75th percentiles (box edges), the most extreme data points not considered outliers (whiskers), and outliers (open circles).

### Protein sequence analysis of NTL9

Functional domains were identified using the programs PROSITE (Falquet et al., 2002) and TMHMM (Krogh et al., 2001) using NTL9 amino acid sequences. Pairwise sequence alignment was obtained with NEEDLE from EMBOSS v6.6.0 (Rice et al., 2000) using the BLOSUM62 matrix method. Multiple sequence alignment was obtained with ClustalW (Thompson et al., 1994), using the BLOSUM matrix method.

## Accession numbers

The Arabidopsis Genome Initiative (AGI) number from The Arabidopsis Information Resource (TAIR) database (http://www.arabidopsis.org/) is as follows: NTL9/PCT (At4G35580), RCI2a (At3G05880), AtPP2CF1 (At3G05640), PXY/TDR (At5G61480), SUC2 (At1G22710), SEOR1 (At3G01680), WOX4 (At1G46480), APL (At1G79430), RTM1 (At1G05760), AHA3 (At5g57350), ANT (At4G37750), EXPA9 (At5G02260), IRX1 (At4G18780), IRX3 (At5G17420), IRX5 (At5G44030), ARF3 (At2G33860), ARF4 (At5G60450), ARF5 (At1G19850), AtERF1 (At4G17500), ERF018 (At1G74930), ERF109 (At4G34410), JAZ7 (At2G34600), JAZ10 (At5G13220), MYC2 (At1G32640), COV1 (At2G20120), HCA2 (At5G62940), MOL1 (At5G51350), RUL1 (At5G05160), JUL1 (At3G15680), JUL2 (At5g25490), and UBC9 (At4g27960).

## Supplemental data

The following materials are available in the online version of this article.

**Supplemental Figure S1.** Isolation of an original *pct.o* mutant.

**Supplemental Figure S2.** Transcript levels of cambium-, phloem-, and xylem-marker genes.

**Supplemental Figure S3.** High resolution of genetic and physical maps of the *PCT* locus.

**Supplemental Figure S4.** Overexpression of the wild-type *NTL9* gene in the *pct* mutant background restored stem vascular growth to wild-type levels.

**Supplemental Figure S5.** *NTL9* splice variants.

**Supplemental Figure S6.** Expression profiles of the *NTL9* gene in different vascular cell types.

**Supplemental Figure S7.** Expression of the *NTL9* transcripts in the aerial parts of 3-week-old plants.

**Supplemental Figure S8.** Expression profiles of phytohormone-regulated and stem secondary growth-associated genes.

**Supplemental Table S1.** Segregation of the *pct* mutant vascular phenotype in primary inflorescence stems with or without the original transgene (*PXYpro: AtPP2CF1*) in F_2_ progenies derived from backcrossing wild-type and *pct.o*.

**Supplemental Table S2.** Primers for the construction of plasmids.

**Supplemental Table S3.** Primers for genotyping.

**Supplemental Table S4.** Cleaved amplified polymorphic sequence (CAPS) and simple sequence length polymorphic (SSLP) markers.

**Supplemental Table S5.** Gene-specific primers for RT-qPCR and RT-PCR analyses.

**Supplemental Table S6.** Gene-specific primers for 3′-RACE.

**Supplemental Method S1.** Construction of plasmids for the establishment of transgenic Arabidopsis plants.

**Supplemental Method S2.** Map-based cloning of the *PCT* gene.

## Supplementary Material

kiac368_Supplementary_Data

## Notes

H.S. and T.T. conceived and designed the study, constructed the plasmids and transgenic plants, performed histological analysis and gene expression analysis, and analyzed the data. R.K.-Y. contributed to the histological and gene expression analyses. N.Mu. and N.Mi. contributed to the data analysis. H.S. was responsible for drafting the manuscript. H.S. and T.T. contributed equally to this study.

The author responsible for distribution of materials integral to the findings presented in this article in accordance with the policy described in the Instructions for Authors (https://academic.oup.com/plphys/pages/general-instructions) is: Hiroki Sugimoto (e1461@mosk.tytlabs.co.jp).
