# Supplementary material for: Transcription factor NTL9 negatively regulates Arabidopsis vascular cambium development during stem secondary growth
Source: Plant Physiol. 2022 Aug 11;190(3):1731–46. doi: 10.1093/plphys/kiac368 (PMC9614505; doi:10.1093/plphys/kiac368)
Supplement: kiac368_Supplementary_Data [file kiac368_supplementary_data.pdf]

Supplemental data

Article title: **Transcription factor NTL9 negatively regulates Arabidopsis vascular cambium development during stem secondary growth**

Authors: Hiroki Sugimoto, Tomoko Tanaka, Nobuhiko Muramoto, Ritsuko Kitagawa-Yogo, and Norihiro Mitsukawa

Supplemental Method S1. Construction of plasmids for the establishment of transgenic Arabidopsis plants.

Arabidopsis genomic DNA and cDNA were used as templates for PCR to construct the plasmids, as described below. The primers used are shown in Supplemental Table S2.

To construct a plasmid expressing *AtPP2CF1* (*At3G05640*) cDNA under the control of the *PXY*/*TDR* promoter (*pPXYpro:AtPP2CF1*), a DNA fragment containing the *PXY*/*TDR* promoter was amplified using PCR with primers P_T21 and P_T22. This PCR fragment was inserted into the HindIII/SalI sites of the *pBI101N2* plasmid (Sugimoto et al., 2014) using the In-Fusion Cloning System (Clontech, Mountain View, CA) according to the manufacturer's instructions to produce *pPXYpro:GUS*. A DNA fragment containing the *AtPP2CF1* cDNA was obtained using PCR with primers P_T18 and P_T19. This PCR fragment was inserted into the SalI/SacI sites of *pPXYpro:GUS* using the In-Fusion Cloning System.

To construct a plasmid expressing the GFP-RCI2a translational fusion gene under the control of the *SUC2* (*At1G22710*) promoter (*pSUC2pro:GFP-RCI2a*), a DNA fragment containing the *SUC2* promoter was obtained using PCR with primers P_T23 and P_T24. This PCR fragment was inserted into the HindIII/SalI sites of the *pBI101N2* plasmid using the In-Fusion Cloning System to produce *pSUC2pro:GUS*. The modified *GFP* DNA fragment with an alanine linker was obtained using PCR with primers P_38 and P_1524 to produce the GFP-Ala DNA fragment. The DNA fragment containing the *RCI2a* (*At3G05880*) cDNA was obtained using PCR with primers P_1525 and P_1526, and was inserted into the *pGEM-T Easy* vector (Promega, Madison, WI) to produce *pRCI2a*. The modified *RCI2a* DNA fragment with an alanine linker (10 consecutive alanines) was obtained using PCR with primers P_1527 and P_1528 to produce the Ala-RCI2a DNA fragment. Two sequential PCRs were performed to produce a DNA fragment containing the GFP-RCI2a translational fusion. The first PCR was performed using GFP-Ala and Ala-RCI2a DNA fragments as templates

without primers. The first PCR product was used as a template for the second PCR using primers P_115 and P_1528. The resulting DNA fragment was inserted into the BamHI/SacI sites of the *pBI121* vector (Clontech, Mountain View, CA) using the In-Fusion Cloning System to produce *p35Spro:GFP-RCI2a*. The modified DNA fragment containing the *GFP-RCI2a* translational fusion region was obtained using PCR with *p35Spro:GFP-RCI2a* as a template and primers P_1531 and P_1528. This PCR fragment was inserted into the SalI/SacI sites of *pSUC2pro:GUS* using the In-Fusion Cloning System.

To construct a plasmid expressing the GUS reporter gene under the control of the *NTL9* promoter (*pNTL9pro:GUS*), a DNA fragment containing the *NTL9* promoter was obtained using PCR with primers P_1393 and P_1394. This PCR fragment was inserted into the SalI/BamHI sites of the *pAtPP2CF1:GUS* plasmid (Sugimoto et al., 2014) using the In-Fusion Cloning System.

To construct a plasmid possessing the *NTL9* genomic region containing both the NTL9 promoter and the entire *NTL9* gene including introns (*pNTL9pro:NTL9g*), a DNA fragment containing the *NTL9* genomic region was obtained using PCR with primers P_1393 and P_1395. This PCR fragment was inserted into the SalI/EcoRI sites of the *pAtPP2CF1:GUS* plasmid (Sugimoto et al., 2014) using the In-Fusion Cloning System.

To construct a plasmid expressing the entire *NTL9* gene, including introns, under the control of the *35S* promoter (*p35Spro:NTL9g*), two sequential PCRs were performed to produce the modified DNA fragment containing the *NTL9* genome. The first PCR was performed using primers P_1407 and P_1408. The first PCR product was used as a template for the second PCR using primers P_115 and P_1408. The resulting DNA fragment containing the *NTL9* genome was inserted into the BamHI/SacI sites of the *pBI121* vector using the In-Fusion Cloning System.

To construct a plasmid expressing *NTL9.1* cDNA under the control of the *35S* promoter (*p35Spro:NTL9.1c*), a DNA fragment containing the *NTL9.1* cDNA was obtained using PCR with primers P_1400 and P_1401. This PCR fragment was inserted into the *pGEM-T Easy* vector to produce *pNTL9.1c*. Two sequential PCRs were performed to produce the modified DNA fragment containing the *NTL9.1* cDNA. The first PCR was performed using *pNTL9.1c* as a template with primers P_1407 and P_1408. The first PCR product was used as a template for the second PCR using primers P_115 and P_1408. The resulting DNA fragment containing the *NTL9.1* cDNA was inserted into the BamHI/SacI sites of the *pBI121* vector using the In-Fusion Cloning System.

Supplemental Method S2. Map-based cloning of the *PCT* gene.

F_2 populations derived from crosses between the *pct* mutant and wild-type Landsberg *erecta* (L*er*)-0 were used for genetic linkage mapping of the *PCT* locus. The *PCT* gene was mapped using sets of cleaved amplified polymorphic sequence (CAPS) and simple sequence length polymorphic (SSLP) markers listed in Supplemental Table S4. To identify the *pct* mutation, the genomic region around the *PCT* locus was compared between wild-type and *pct* mutants using whole-genome sequencing with next-generation sequencing, and one base insertion (a nucleotide A) in the coding region of *NTL9* (*At4G35580*) was identified.

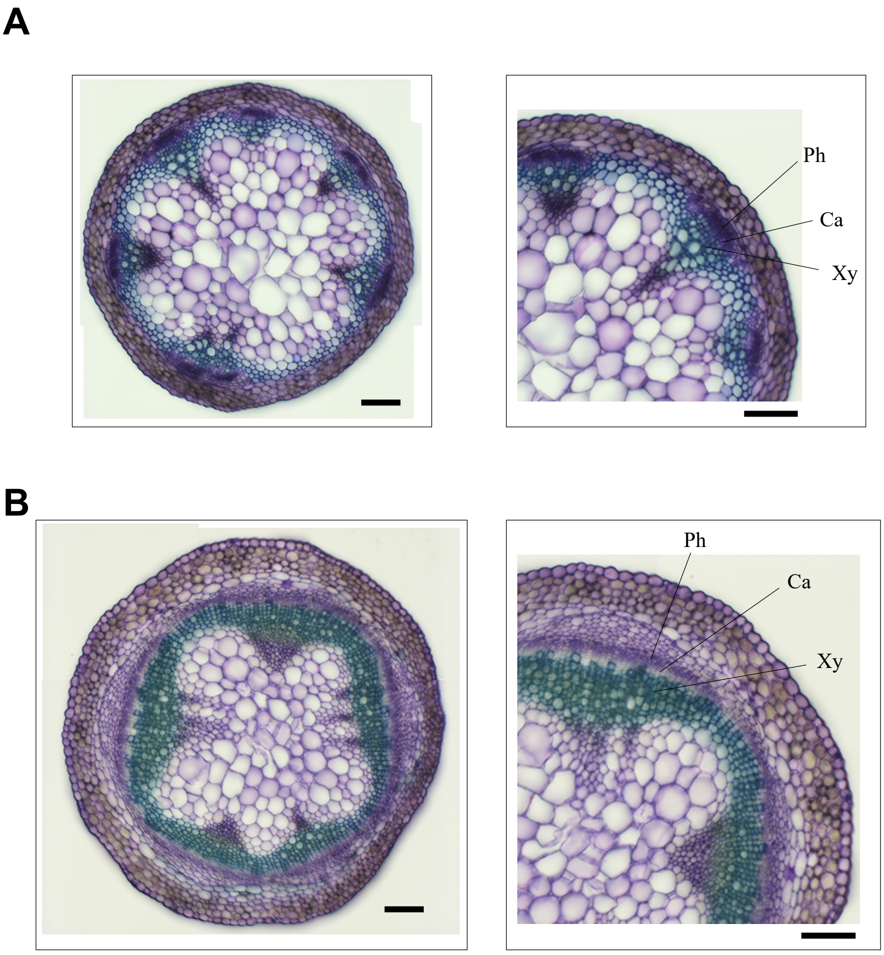

Supplemental Figure S1 Isolation of an original *pct.o* mutant. Transverse primary inflorescence stem sections from wild-type (A) and *pct.o* mutant (B) plants. Basal regions (20 mm from the rosette base) of primary inflorescence stems from 9-week-old plants were used for observations. Cross-sections were 100-μm-thick and stained with toluidine blue. Ca, cambium; Ph, phloem; Xy, xylem. Scale bars, 100 μm.

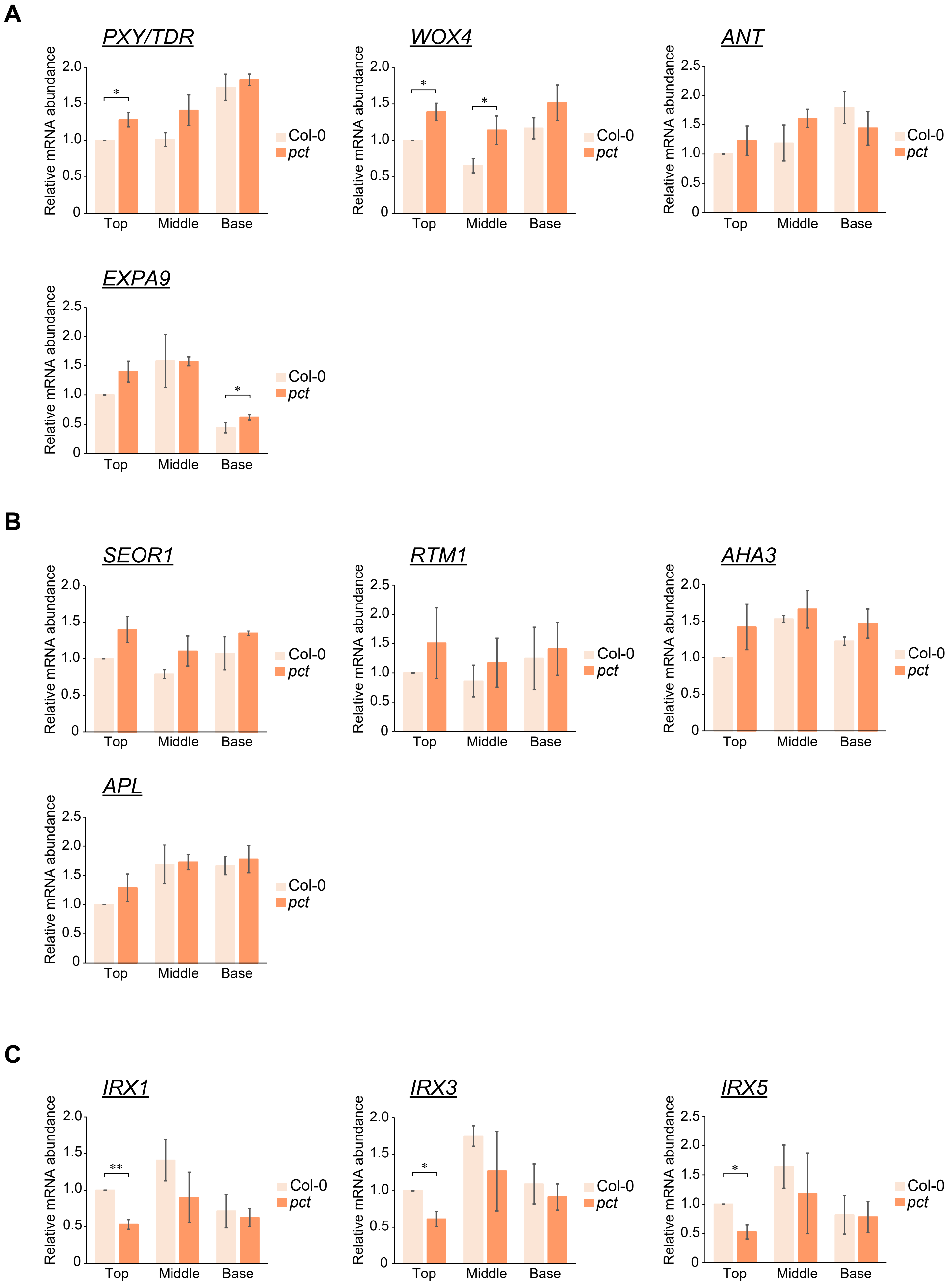
A
PXY/TDR
WOX4
ANT
EXPA9
B
SEOR1
RTM1
AHA3
APL
C
IRX1
IRX3
IRX5
Relative mRNA abundance
Top
Middle
Base
Col-0
pct
*
**

Supplemental Figure S2 Transcript levels of cambium-, phloem-, and xylem-marker genes. Total RNA was isolated from three different regions (top, middle, and base) of 9-week-old inflorescence stems and subjected to RT-qPCR. The expression ratio of each gene to the *UBC9* gene was calculated for each sample. The values of samples for the top regions of the wild-type were set at 1 and used to determine the relative abundance for the other samples. A, Transcript levels of cambium-marker genes. B, Transcript levels of phloem-marker genes. C, Transcript levels of xylem-marker genes. Values represent the mean ± S.D. of three biological and technical replicates (*, $P < 0.05$; **, $P < 0.01$; Welch' s *t*-test).

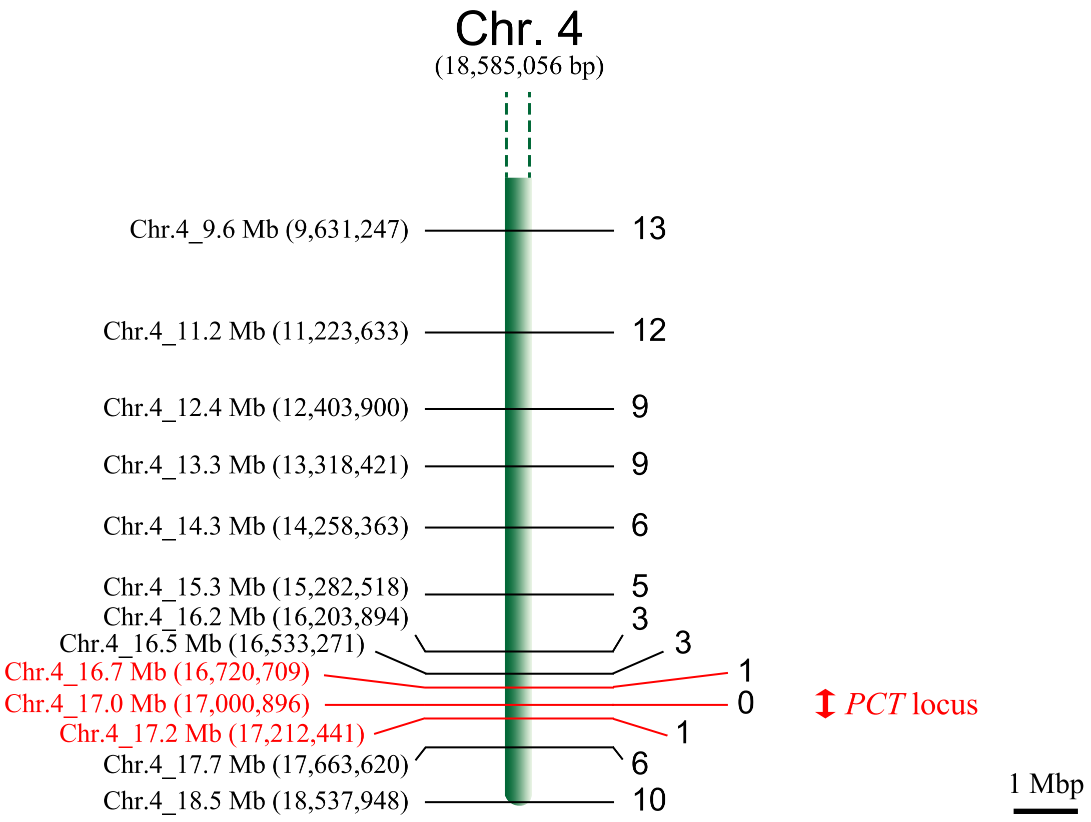

Supplemental Figure S3 High resolution of genetic and physical maps of the *PCT* locus. The genetic and physical linkage map representing the relative position of *PCT* to the CAPS and SSLP markers on the long arm of chromosome 4. The left parts show CAPS and SSLP markers (Supplemental Table S3). The nucleotide position on chromosome 4 is indicated in parenthesis. To identify the *PCT* gene, we mapped *PCT* to chromosome 4, delimited to a locus within a 500-kb region between the markers Chr.4_16.7 Mb and Chr.4_17.2 Mb using 72 F_2 chromosomes. The values on the right are the numbers of recombinants in the intervals between the *PCT* locus and each marker.

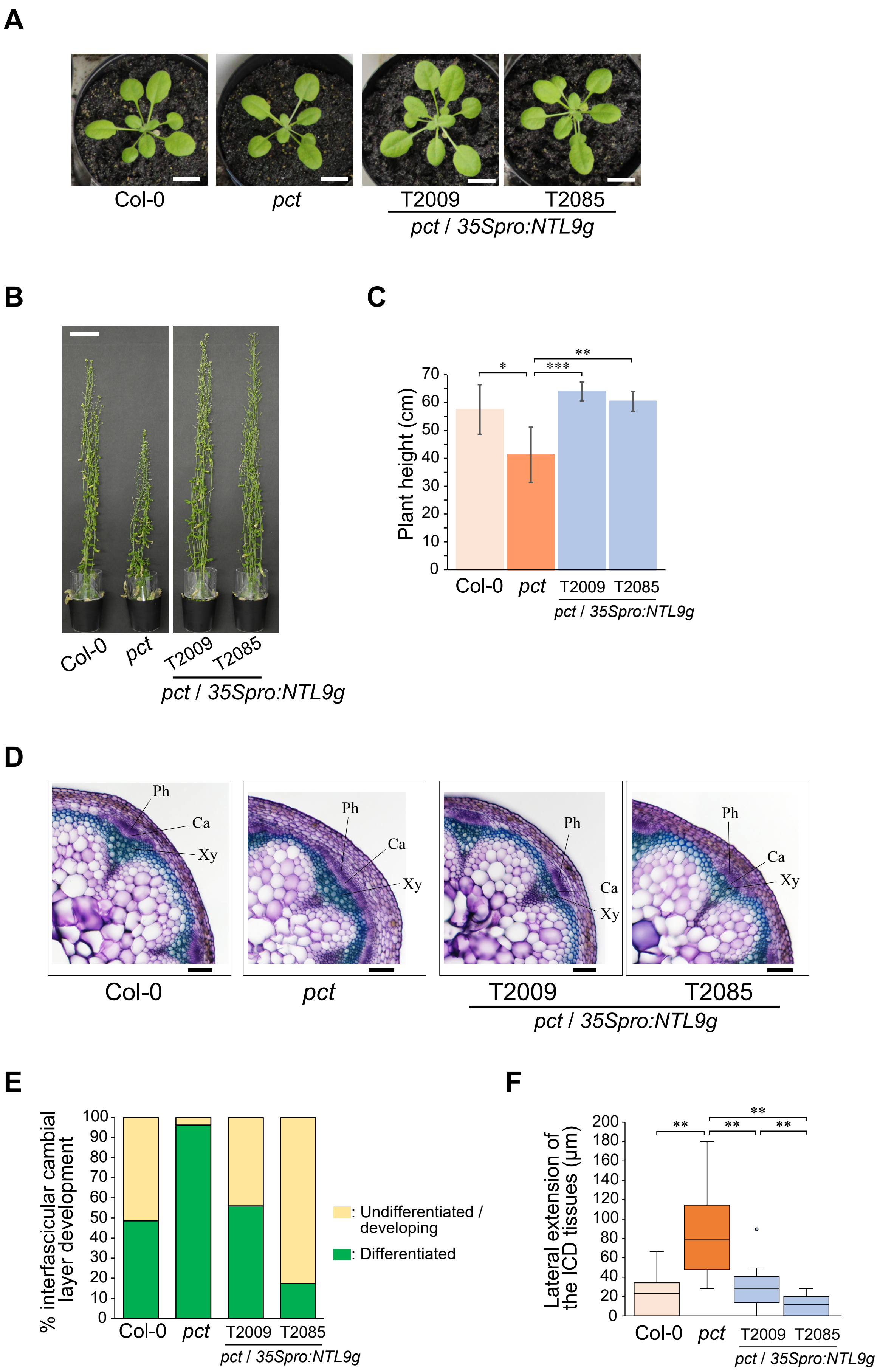

Supplemental Figure S4 Overexpression of the wild-type *NTL9* gene in the *pct* mutant background restored stem vascular growth to wild-type levels. A and B, The 25-d-old (A) and 9-week-old (B) *pct*/*35Spro:NTL9g* line plants. Two independent *pct*/*35Spro:NTL9g* line plants (T2009 and T2085) were examined. Scale bars, 10 mm (A); 50 mm (B). C, Height of 8-week-old plants. Two independent

pct/*35Spro:NTL9g* line plants (T2009 and T2085) were examined. Values represent the mean ± S.D. *, $P < 0.05$; **, $P < 0.01$; ***, $P < 0.001$; one-way ANOVA followed by the Tukey–Kramer test; $n = 4$ for Col-0, $n = 6$ for *pct*, $n = 5$ for T2009, $n = 5$ for T2085. D, Transverse primary inflorescence stem sections from *pct*/*35Spro:NTL9g* line plants. Basal parts (20 mm from the rosette base) of primary inflorescence stems in 8-week-old plants were used for observations. Two independent *pct*/*35Spro:NTL9g* line plants (T2009 and T2085) were examined. Enlarged images of vascular bundles are shown. Cross-sections were 100-μm-thick and stained with toluidine blue. Ca, cambium; Ph, phloem; Xy, xylem. Scale bars, 100 μm. E, Differentiation levels of the basal regions of inflorescence stems. Differentiation levels were determined as a percentage of the number of differentiated interfascicular regions, based on whether the interfascicular cambium-derived (ICD) tissues interconnected two adjacent primary vascular bundles, to total interfascicular regions analyzed. Two independent *pct*/*35Spro:NTL9g* line plants (T2009 and T2085) were examined. F, Quantitative analysis of cambial activity. The cambial activity was determined as a lateral extension of ICD tissues at basal regions (20 mm from the rosette base) of primary inflorescence stems of 8-week-old plants. See Figure 1, F and G for the definition of ICD tissues. Two independent *pct*/*35Spro:NTL9g* line plants (T2009 and T2085) were examined. See also Materials and methods for boxplot definition. **, $P < 0.01$; Kruskal–Wallis test followed by the Steel–Dwass test; $n = 35$ for Col-0, $n = 27$ for *pct*, $n = 25$ for T2009, $n = 23$ for T2085.

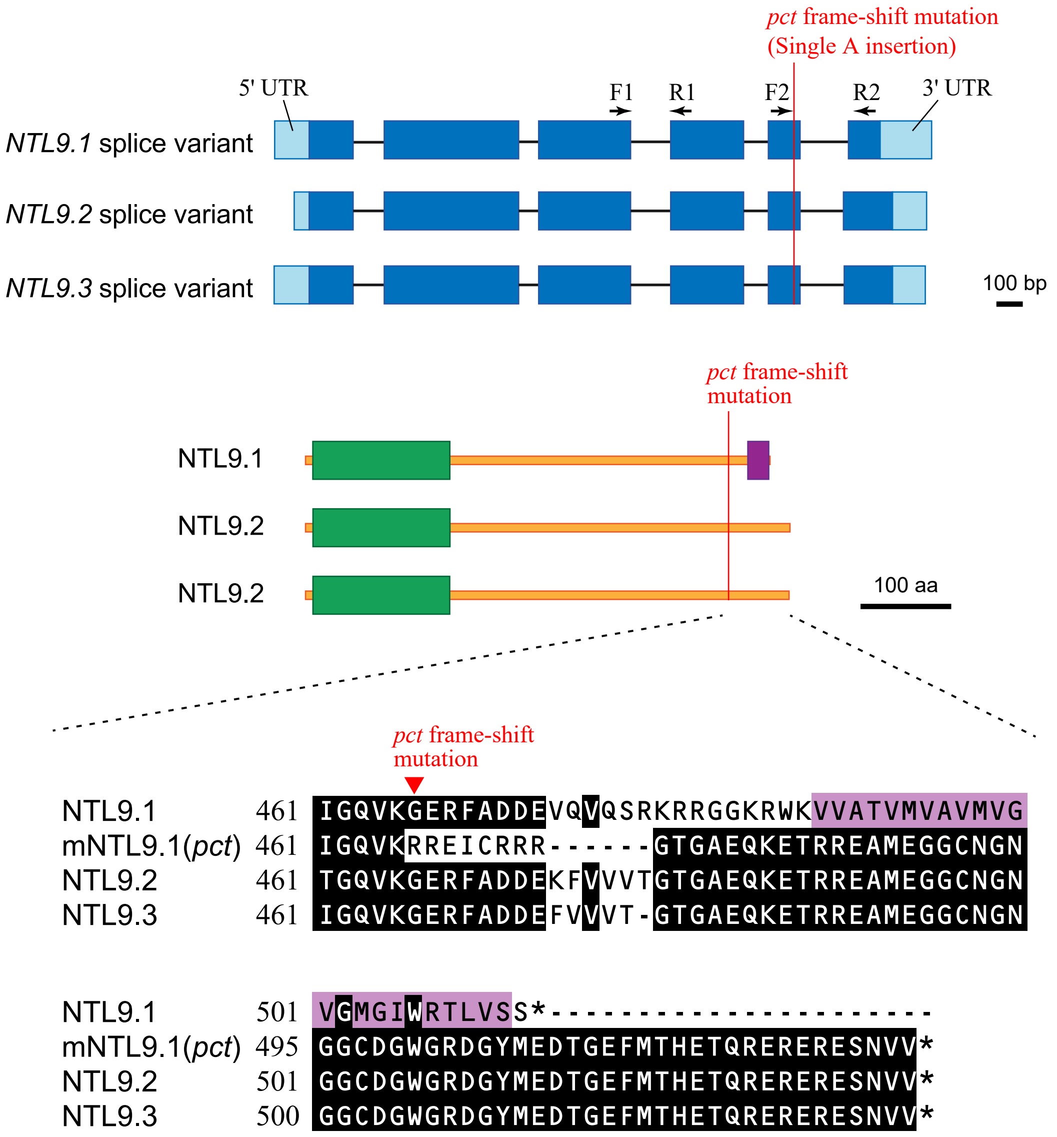

Supplemental Figure S5 *NTL9* splice variants. The upper part shows *NTL9* splice variant (*NTL9.1–3*) structures, with boxes representing exons and thin lines representing introns. The open reading frame of each *NTL9* splice variant is depicted as blue boxes. The position of a single adenine nucleotide insertion in the *pct* mutant is indicated. Two primer set positions are indicated by arrows. Primers (F1 and R1) spanning exons 3 to 4 were synthesized and used; these were the DNA fragments identical in length across all splice variants of *NTL9* gene. Primers (F2 and R2) spanning exons 5 to 6 were used to distinguish between each splice variant of *NTL9* gene. The central part shows the protein structure encoded by each *NTL9* splice variant. The NAC transcriptional domain and transmembrane domain are depicted as green and purple boxes, respectively. The start position of the frameshifted sequence caused by *pct* mutation is indicated. Functional domains were identified using the programmes PROSITE (Falquet et al., 2002) and TMHMM (Krogh et al., 2001). The bottom part shows alignments of C-terminal sequences of wild-type and mutant NTL9 variants. The corresponding residues in transmembrane domain of NTL9.1 are boxed in purple. In mutated NTL9.1, resulting from the *pct* mutation (mNTL9.1), a single adenine nucleotide insertion would be expected to result in mistranslated sequence of C-terminus of NTL9.1, which is the dominant form of NTL9 variants, but might be instead replaced with newly translated amino acids. Multiple sequence alignment was obtained with ClustalW (Thompson et al., 1994). The residues in black boxes are identical in at least three of the four NTL9 variants. Amino acid numbers for each NTL9 variant are shown on the left. The asterisks represent stop codons.

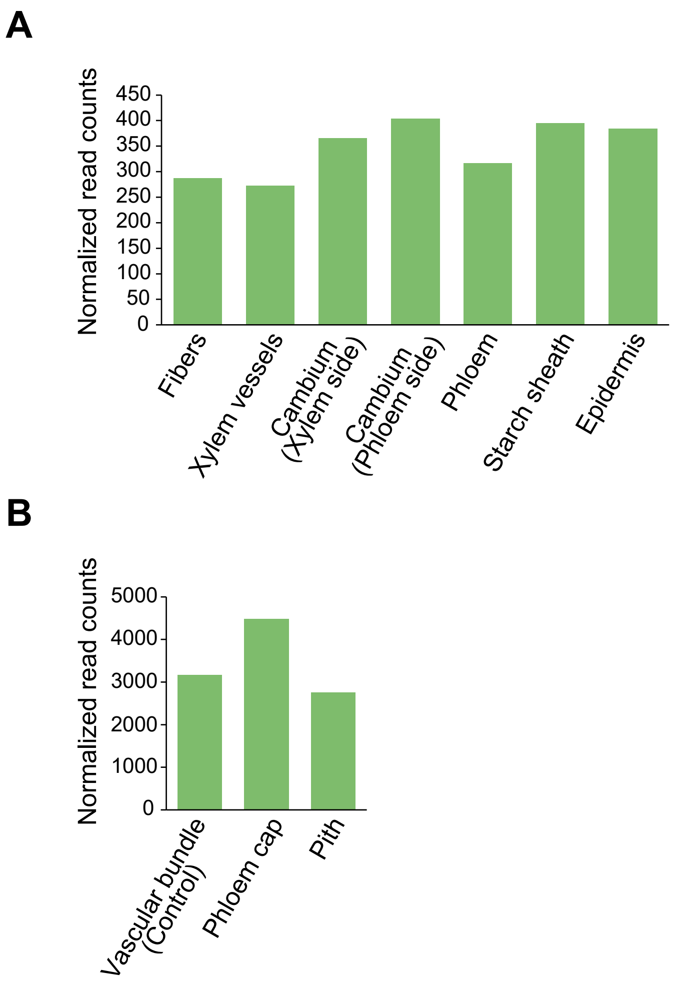

Supplemental Figure S6 Expression profiles of the *NTL9* gene in different vascular cell types. Average normalized read count values were obtained from the Arabidopsis fluorescence-activated nucleus sorting (FANS)– and laser capture microdissection (LCM)–derived transcriptome data website (https://arabidopsis-stem.cos.uni-heidelberg.de/; Shi et al., 2021) using *At4G35580* as the query. A, *NTL9* gene-expression profile based on the FANS-derived RNA-seq datasets. B, *NTL9* gene-expression profile based on the LCM-derived RNA-seq datasets.

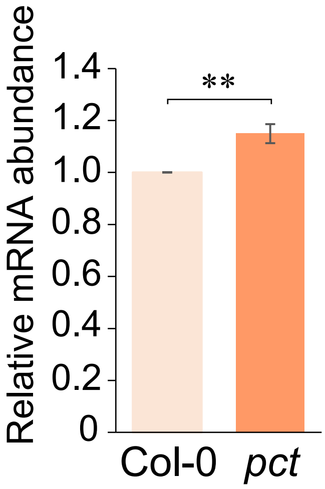

Supplemental Figure S7 Expression of the *NTL9* transcripts in the aerial parts of 3-week-old plants. Total RNA was isolated from the aerial parts of wild-type and *pct* mutant plants and subjected to RT-qPCR. Primers (F1 and R1), which span exons 3 to 4 of the *NTL9* gene (shown in Supplemental Figure S5), were used. The expression ratio of *NTL9* gene to the *UBC9* gene was calculated for each sample. The values for wild-type samples were set at 1 and used to determine the relative abundance for the *pct* mutant samples. Values represent the mean ± S.D. of four biological and three technical replicates (**, $P < 0.01$; Welch’ s *t*-test).

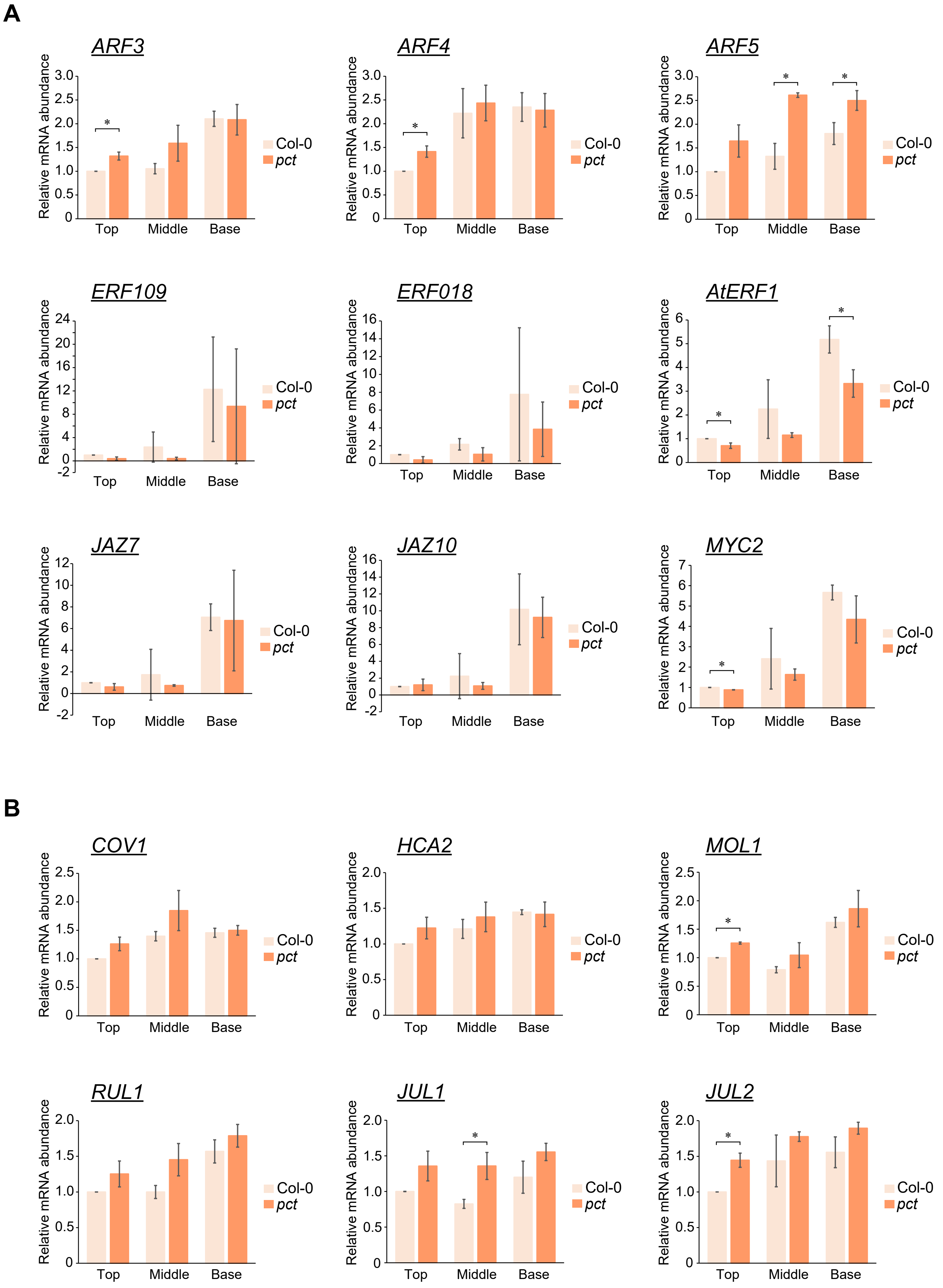
A
ARF3
ARF4
ARF5
ERF109
ERF018
AtERF1
JAZ7
JAZ10
MYC2
B
COV1
HCA2
MOL1
RUL1
JUL1
JUL2
Relative mRNA abundance
Top
Middle
Base
Col-0
pct
*

Supplemental Figure S8 Expression profiles of phytohormone-regulated and stem secondary growth-associated genes. Total RNA was isolated from three different regions (top, middle, and base) of 9-week-old inflorescence stems and subjected to RT-qPCR. The expression ratio of each gene to the *UBC9* gene was calculated for each sample. The values of samples for the top regions of the wild-type were set at 1 and used to determine the relative abundance for the other samples. A, Transcript levels of phytohormone (auxin, ethylene, and jasmonate)-regulated genes. B, Transcript levels of stem secondary growth-associated genes. Values represent the mean ± S.D. of three biological and technical replicates (*, $P < 0.05$; Welch’ s *t*-test).

Supplemental Table S1 Segregation of *pct* mutant vascular phenotype in primary inflorescence stems with or without the original transgene (*PXYpro:AtPP2CF1*) in F_2 progenies derived from backcrossing wild-type and *pct.o*.

Stem phenotye	Wild-type		*pct* mutant			
Transgene [a]	+	-	+	-	Total	χ^{2} [b]
F_2 progenies	16	9	14	1	40	3.33

[a] +/- indicate plants with or without the transgene, respectively.
[b] χ^2 value for wild-type : mutant stem phenotype segregation of 3 : 1 was calculated using two phenotypic classes (one degree of freedom). The 95% confidence limit for rejecting the expected 3 : 1 segregation is ≥3.84 and the 99% limit is ≥6.63.

Supplemental Table S2 Primers for the construction of plasmids.

Primer Names	Primer Sequences (5' to 3')
P_38	AGGATCCCCGGGTGGTCAGTCCCTTATGGTGAGCAAGGGCGAGGA
P_115	TTGGAGAGAACACGGGGGACTCTAGAGGATCCCCGGGTGGTCAGTC
P_1393	GCGCCTTAATTAAACTAGTCTCGAGGTCGACGGTCAGATTATGAT
P_1394	GTAACATAAGGGACTGACCACCCGGGGATCCAATCCTCTTCTTTAGGGTTTC
P_1395	ACGACGTTGTAAAACGACGGCCAGTGAATTCAGACGATTTCAAAA
P_1400	AGAAAAAAAAGCAGAGAGTGTATTCAAAGT
P_1401	TAATTAAGATGTTGGTACATTTAATCTTGA
P_1407	CCCCGGGTGGTCAGTCCCTTATGGGTGCTGTATCGATGGA
P_1408	TTGAACGATCGGGGAAATTCGAGCTCTAATTAAGATGTTGGTACAT
P_1524	TGCAGCTGCAGCTGCAGCTGCAGCTGCAGCCTTGTACAGCTCGTCCATG; the alanine linker site is underlined.
P_1525	ATGAGTACAGCTACTTTCGT
P_1526	AATGGTTAATGGTGGTCCT
P_1527	CAGCTGCAGCTGCAGCTGCAATGAGTACAGCTACTTTCGT; the alanine linker site is underlined.
P_1528	TTGAACGATCGGGGAAATTCGAGCTCAATGGTTAATGGTGGTCCT
P_1531	ACTTTCTTGGTTTGTCAAATGTCGACATGGTGAGCAAGGGCGAGGA
P_T18	TTCTTTCTAAAAGCTACGGTCGACATGGGACATTTCTCTTCC
P_T19	GAACGATCGGGGAAATTCGAGCTCCTATAGAGATGGCGACGA
P_T21	TATGACCATGATTACGCCAAGCTTAAGAAGTTGATTTTGGAC
P_T22	ACCCGGGGATCCTCTAGAGTCGACCGTAGCTTTTAGAAAGAA
P_T23	TATGACCATGATTACGCCAAGCTTACGCAAACTAACTACAAC
P_T24	ACCCGGGGATCCTCTAGAGTCGACATTTGACAAACCAAGAAA

Supplemental Table S3 Primers for genotyping.

Primer Sequences (5' to 3')	Restriction Enzymes Used	Expected Sizes of DNA Fragments
For the identification of T-DNA (*pPXYpro:AtPP2CF1*) insertion		
AAGCCAGACCATTAGACGCA<br>AGGGACCATGTCCATCGAAT	—	497 bp
For derived cleaved amplified polymorphic sequence (dCAPS) for the identification of *pct* mutation		
CTCAAATAAAGCTCAGGGCGCGGGGAACTATAGGCCCTGT<br>TGGGGAGAGACGACGATTGA	EcoNI	Col-0 : 588 bp<br>*pct* : 629 bp

Supplemental Table S4 Cleaved amplified polymorphic sequence (CAPS) and simple sequence length polymorphic (SSLP) markers.

Markers	Marker Type	Nucleotide Positions on Chromosome 4	Primer Sequences (5' to 3')	Restriction Enzymes Used	Expected Sizes of DNA Fragments
Chr.4_9.6 Mb	CAPS	9,631,247	GGACGTAGAATCTGAGAGCTC<br>GGTCATCCGTTCCCAGGTAAAG	HindIII	Col-0: 565 bp<br>L*er*-0: 474 bp
Chr.4_11.2 Mb	SSLP	11,223,633	TTGGACCGTCCAGAAAAGGG<br>TGGGTCTTGAACCGGTCTTG	—	Col-0: 218 bp<br>L*er*-0: 292 bp
Chr.4_12.4 Mb	CAPS	12,403,900	GAAGGTCGGCGTAAGAAGATCT<br>TATCCTTGAACGCTGGGCTGCA	MseI	Col-0: 202 bp<br>L*er*-0: 303 bp
Chr.4_13.3 Mb	SSLP	13,318,421	AACACCGTCCTTGATCTTAGAG<br>AAGTAGCTACCTCGGTATTGGT	—	Col-0: 150 bp<br>L*er*-0: 198 bp
Chr.4_14.3 Mb	SSLP	14,258,363	CCGCATGATAAGCTAAAGTCGA<br>ACTTGAAAACACTTACCGTGAAGT	—	Col-0: 124 bp<br>L*er*-0: 164 bp
Chr.4_15.3 Mb	CAPS	15,282,518	GGCGGCACTGGTGGTGTAGG<br>GTTGTCCCCTGTTAAAAGGAGCC	MnlI	Col-0: 188 bp<br>L*er*-0: 328 bp
Chr.4_16.2 Mb	SSLP	16,203,894	CATGGGGGCAATGTATTTTACAC<br>TAGAGTTTCACTACTTCCAGCAC	—	Col-0: 161 bp<br>L*er*-0: 120 bp
Chr.4_16.5 Mb	CAPS	16,533,271	CCAGTTGGTCGACCTAAACCCC<br>AATTCACCACGATCTACTAATCTGC	RsaI	Col-0: 161bp<br>L*er*-0: 300 bp
Chr.4_16.7 Mb	SSLP	16,720,709	GTTAGCATGAGACGCTCCACAT<br>CACCGGCAATTTTCTCATCCAG	—	Col-0: 157 bp<br>L*er*-0: 122 bp
Chr.4_17.0 Mb	SSLP	17,000,896	TGAGAGAAAGACTTGTGAGTCTCTT<br>AACCACAAGACGCTGCCGTT	—	Col-0: 155 bp<br>L*er*-0: 193 bp
Chr.4_17.2 Mb	SSLP	17,212,441	GTTTACAATCGCTAACTACTAATG<br>CTATGGAGGTTTCCAAAACTTAAC	—	Col-0: 162 bp<br>L*er*-0: 211 bp
Chr.4_17.7 Mb	SSLP	17,663,620	AGTTACTTGATCAAACCTTGATTAC<br>TCGACATGTTTCTTAAGGTACAAG	—	Col-0: 215 bp<br>L*er*-0: 128 bp
Chr.4_18.5Mb	CAPS	18,537,948	AGAGAGAATGAGAAATGGAGG<br>CAAGTGACCTGAAGAGTATCG	MboII	Col-0: 1,082 bp<br>L*er*-0: 553 bp

Supplemental Table S5 Gene-specific primers for RT-qPCR and RT-PCR analyses.

Genes	Primer Sequences (5' to 3')	References
NTL9 (*At4G35580*)	For RT-qPCR analysis<br>F1: AGTCTGCAGCACCAGAGGATG<br>R1: CACTTGGCCAACCAGAAGCTC<br>For RT-PCR analysis<br>F2: TCAAATAAAGCTCAGGGCGCG<br>R2: CCTCGTCTCTTTCTGCTCTGC	this study
APL (*At1G79430*)	ACCAAGTCCTCGACCATCACA<br>CTCCGACAAAGAATCCAAATCC	Guo et al., 2009
RTM1 (*At1G05760*)	ATTGTAAACTGGGACGAAGGAT<br>CGTACTCGCTAGTGTTGGTATTG	Guo et al., 2009
AHA3 (*At5G57350*)	GCTGGTATGGATGTTCTGTGC<br>GGTTCGCTTATCAACTGGATT	Guo et al., 2009
SEOR1 (*At3G01680*)	TCTCACGGCCTTGGTTCATC<br>ATCGACCCCAACCAAGTTCC	Cho et al., 2018
ANT (*At4G37750*)	GATGTAGCAGCAATTAAGTTCCG<br>GAGCGGTTTGGTCTTCAGTATT	Guo et al., 2009
EXPA9 (*At5G02260*)	TGATCTCGCTATGCCTATGTTT<br>TGACCAGCACCAAGTTGAAGTA	Guo et al., 2009
PXY/TDR (*At5G61480*)	CCGGTTCGTACGGTTACATT<br>ACGACCTACCCATGCTTTTG	Agusti et al., 2011
WOX4 (*At1G46480*)	TGGTGGAGAAGGAGGAGT<br>TCATGACTTCATCTCCCTTCAGGA	Agusti et al., 2011
IRX1 (*At4G18780*)	CATCCCAACGCTATCAAACCTA<br>GCTGAGACACCTCCAATAACCC	Guo et al., 2009
IRX3 (*At5G17420*)	CAAAGGGTCCTAAACGTCCA<br>ATGGATGATTGCCCAAATGT	Cho et al., 2018
IRX5 (*At5G44030*)	TCTGGGTGATTGGCGGTG<br>GTCGGAGGGATGAGAAGGGT	Guo et al., 2009
HCA2 (*At5G62940*)	CACATTTTGGTGGGATGATGGG<br>AAGCAAACCGCCATACTTGC	Miyashima et al., 2019
COV1 (*At2G20120*)	CCGAGGAGGAACTTTGCTG<br>CACCCGAAACAACAATCTCAA	Guo et al., 2009
MOL1 (*At5G51350*)	ATGCCGGTGGATTGATGATA<br>GGACGGTCAGACTGGTCACT	Agusti et al., 2011
RUL1 (*At5G05160*)	CCATTGGCTACAATGCTCCT<br>GAACCCATCTCGGAAGATCA	Agusti et al., 2011
JUL1 (*At3G15680*)	CACCTGCTTCAAATGCGGCA<br>GGGGACTCTAGACCGCATAA	this study
JUL2 (*At5G25490*)	TGTTTCAAGTGTGGTGCCGC<br>ACCACTACTGCTGCCGCCAA	this study
ARF3 (*At2G33860*)	ATCATCGGCAGCAGCACG<br>TGGTAAGCAAGACGGAAGAGG	Liu et al., 2018
ARF4 (*At5G60450*)	GCTCGCTTAAATCATTCCC<br>ACTTGACTTGTTGGCTTGGTA	Liu et al., 2018
ARF5 (*At1G19850*)	GGTGCTTCGTCGCCTGTA<br>TGGGAGGATAGAATGAATGGTT	Liu et al., 2018
JAZ7 (*At2G34600*)	ATCCCAAACAATTCGACTCG<br>GGAAGTTGCTTGAATCCGAA	Yu et al., 2016
JAZ10 (*At5G13220*)	ATGTCGAAAGCTACCATAGAACTCG<br>GCCGATGAATCGGAATTGTTTCCAGTGG	Sehr et al., 2010
MYC2 (*At1G32640*)	AAGCTTCCGTCGTGAAAGAA<br>CTTCTCTACCGTTTGCTGGC	Yu et al., 2016
AtERF1 (*At4G17500*)	CGGTTCGAATCAAGTCCAAG<br>CACCGTTCTCCTCTTCTTCG	Etchells et al., 2012
ERF018 (*At1G74930*)	TTGATTCCTTCTCCGACGAC<br>TCCTCCGTAATCTTCGATGG	Etchells et al., 2012
ERF109 (*At4Gg34410*)	CAGTTGAAGCAGAGCAATGG<br>CCATTCCCAAAATCCATCAT	Etchells et al., 2012
UBC9 (*At4Gg27960*)	TCACAATTTCCAAGGTGCTGC<br>TCATCTGGGTTTGGATCCGT	Sugimoto et al., 2014

Supplemental Table S6 Gene-specific primers for 3'-RACE.

Primer Names	Positions in *NTL9.1* Splice Variant	Primer Sequences (5' to 3')
P_F19	Exons 4/5	CGAGGCCAAAGAAGCTGCAG
P_F20	Exon 5	CCTCAAATAAAGCTCAGGGC
P_F21	Exon 6	GGACACTGGTGAGTTCATGA
